# Mapping the Interactome of a Major Mammalian Endoplasmic Reticulum Heat Shock Protein 90

**DOI:** 10.1371/journal.pone.0169260

**Published:** 2017-01-05

**Authors:** Feng Hong, Saleh Mohammad Rachidi, Debbie Lundgren, David Han, Xiu Huang, Hongyu Zhao, Yayoi Kimura, Hisashi Hirano, Osamu Ohara, Heichiiro Udono, Songdong Meng, Bei Liu, Zihai Li

**Affiliations:** 1 Department of Microbiology & Immunology, Medical University of South Carolina, Charleston, United States of America; 2 Center for Vascular Biology, University of Connecticut Health Center, Farmington, Connecticut, United States of America; 3 Department of Epidemiology and Public Health, Yale University, School of Medicine, New Haven, Connecticut, United States of America; 4 Laboratory for Immunogenomics, RIKEN Research Center for Allergy and Immunology, Tsurumi-ku, Yokohama, Kanagawa, Japan; 5 Graduate School of Nanobioscience, Yokohama City University, Tsurumi-ku, Yokohama, Kanagawa, Japan; 6 Kazusa DNA Research Institute, Kisarazu, Chiba, Japan; 7 Department of Immunology, Okayama University Graduate School of Medicine, Dentistry and Pharmaceutical Sciences, Japan; 8 CAS Key Laboratory of Pathogenic Microbiology and Immunology, Institute of Microbiology, Chinese Academy of Sciences (CAS), Beijing, China; Universite de Geneve, SWITZERLAND

## Abstract

Up to 10% of cytosolic proteins are dependent on the mammalian heat shock protein 90 (HSP90) for folding. However, the interactors of its endoplasmic reticulum (ER) paralogue (gp96, Grp94 and HSP90b1) has not been systematically identified. By combining genetic and biochemical approaches, we have comprehensively mapped the interactome of gp96 in macrophages and B cells. A total of 511 proteins were reduced in gp96 knockdown cells, compared to levels observed in wild type cells. By immunoprecipitation, we found that 201 proteins associated with gp96. Gene Ontology analysis indicated that these proteins are involved in metabolism, transport, translation, protein folding, development, localization, response to stress and cellular component biogenesis. While known gp96 clients such as integrins, Toll-like receptors (TLRs) and Wnt co-receptor LRP6, were confirmed, cell surface HSP receptor CD91, TLR4 pathway protein CD180, WDR1, GANAB and CAPZB were identified as potentially novel substrates of gp96. Taken together, our study establishes gp96 as a critical chaperone to integrate innate immunity, Wnt signaling and organ development.

## Introduction

Heat shock proteins (HSPs) are a class of functionally related molecular chaperones involved in numerous processes, such as protein folding, assembly and transport, peptide trafficking and antigen presentation [1–5]. The expression is increased when cells) are exposed to superphysiological temperatures or other stress including infection and inflammation [6].

gp96, also known as endoplasmin, grp94 and ERp99, is one of the endoplasmic reticulum HSPs that is encoded by the *HSP90B1* gene in humans [7–10]. gp96 shares ~50% homology at the amino acid level with its cytosolic HSP90, with a similar domain organization consisting of an N-terminal ATP-binding domain, a charged middle domain and a C-terminal homodimerization domain [11–13]. gp96 is believed to be one of the key downstream chaperones that mediates the ER unfolded protein response (UPR), which is crucial for maintaining protein homeostatsis [11, 14]. UPR dysregulation has been implicated in a variety of diseases including obesity, diabetes and neurodegenerative diseases [15]. However, the role of gp96 remains unclear in these diseases due to incomplete knowledge of the gp96 interactors. Recent genetic studies demonstrated that gp96 is critical for post-translational folding and cell surface expression of two families of proteins: integrins and Toll-like receptors (TLRs) [16–21], suggesting that gp96 may play important roles in immune response. Earlier work suggest that other proteins, such as ErbB2, immunoglobulin heavy chain and light chain, and thyroglobulin are gp96 client proteins [11]. In the case of immunoglobulin folding, the role of gp96 was not substantiated by a genetic study [18]. Unlike its cytosolic counterpart HSP90 that chaperones up to 10% of cytosolic proteins, the known gp96 client proteins remain limited. Moreover, HSP90 has been found to work with a number of co-chaperones, including p50/Cdc37, p23, AHA1, Cpr6 and Cpr7 [22–25]. These cofactors help to link HSP90 to clients, stabilize the ATP bound status of HSP90, and enhance HSP90 ATPase activity. However, so far, only CNPY3, an ER protein, has been identified as a gp96 cofactor, which co-chaperones TLRs with gp96 [21].

We undertook an integrated, large-scale proteomic and genomic study in wild type and conditional knockout (KO) gp96 mice/cells aimed at comprehensively mapping the interactome of gp96. We combined four complementary experimental strategies. First, we use isotope-coded affinity tag (ICAT) technique to detect the reduced protein level in bone marrow derived macrophage (BMDM) from gp96 KO mice, comparing with BMDM from wild type (WT) mice. Second, we performed the comparative and quantitative 2-D gel electrophoresis, coupled with mass spectrometry, and demonstrated the reduction of proteins on the plasma membrane from gp96 KO BMDM. Third, we used mass spectrometry to quantitatively compare the protein expression level between WT and KO mice. The proteins with decreased levels in gp96 KO B cells, comparing as to gp96 WT B cells, are potential interactors of gp96. Lastly, we tagged gp96 and carried out immunoprecipitation (IP) by using Tag-specific antibodies. Gp96 and its associated proteins were then identified by mass spectrometry. Collectively, these efforts identified 511 proteins that are reduced in gp96 KO cells, many of which suggest possible links of gp96 with previously unknown roles in metabolic process, cytoskeleton, translation, transport and Wnt signaling.

## Materials and Methods

### Mice and genotyping

Conditional gp96-deficient mice were described previously [17]. Genotyping was performed by polymerase chain reaction (PCR) amplification of mouse tail genomic DNA to differentiate *WT* (561 bp) from floxed HSP90b1 allele (638 bp) (forward primer: 5′-TGCCAGAGACTACAATTCCCAGCA-3′; reverse primer: 5′-AAACACGAACT CACCAATCGTGCC-3′), to determine whether floxed HSP90b1 underwent successful cre-mediated recombination (440 bp) (forward primer: 5′-AGCAAGGGCCAAGCTACGCAACTG-3′; reverse primer: 5′-CAGGAAGGCTTCCC-CCGG-3′), to identify CD19-cre (715 bp) (forward primer: 5′-AACCAGTCAACACCCTTCC-3′; reverse primer: 5′-TCAGCTACAC CAGAGACGG-3′), and to confirm the presence of *WT* CD19 locus (450 bp) (forward primer: 5′-AACCAGTCAACACCCTTCC-3′; reverse primer: 5′-CCAGACTAGATACAGACCAG-3′). LysM genotyping primers are 5'-CCC AGA AAT GCC AGA TTA CG-3', 5'-CTT GGG CTG CCA GAA TTT CTC-3’ and 5'-TTA CAG TCG GCC AGG CTG AC-3'. LysM^Cre^ and CD19^cre^ mice were purchased from The Jackson Laboratory (Bar Harbor, ME).

Mice were bred, maintained and euthanized for bone marrow and B cell isolation according to the established guidelines for the care and use of laboratory animals of the National Institutes of Health and an approved protocol by Medical University of South Carolina Institutional Animal Care and Use Committee. No *in vivo* experiment was performed in this study.

### Cell lines and plasmids

WT and gp96 mutant preB cell lines were obtained from Brian Seed (Boston, MA). gp96 constructs in MigR vector were previously described [17].

### 2-DE gel image quantitation and protein digestion for MS analysis

For 2-DE analysis, triplicate gels from WT and KO group were run. Briefly, protein (50 μg) was loaded for the preparative gel, which was simultaneously run with the analytical gels under the same experimental conditions. The 2-DE gel was then fixed and stained with Sypro Ruby dye after gel electrophoresis. The scanned 2-DE gel images were imported into Decyder software version 6.5 for protein quantitation analysis. Protein spots showing 1.2-fold change or greater, either increase or decrease, and with a p value < 0.05, (t-test) were selected. Differentially expressed protein spots were excised from the gel, digested with trypsin, and analyzed using MS for protein identification [26]. The obtained MS/MS data were subjected to database searches using the MASCOT program (Matrix Science Ltd., London, UK) with the following parameters: two missed cleavage sites and a peptide and MS/MS mass tolerance setting of ± 100 ppm and ± 0.3 Da, respectively, for MS/MS Ions Search. The database used for this search consisted of amino acid sequences of *Mus musculus* proteins, which were retrieved from a subset of the International Protein Index (IPI) database (var. 3.29) or the non-redundant protein database. Chemical modifications such as oxidation of Met, N-terminal acetylation (Protein) and propionamide of Cys were taken into consideration for the database searches.

### Isotope-coded affinity-tag-based protein profiling

Proteins were labeled with isotopically light-(12C, for WT BMDMs) or heavy- (13C, for KO BMDMs) ICAT reagents following the manufacturer’s protocol (Applied Biosystems, Foster City, CA). Corresponding isotopically light- and heavy-labeled samples were then combined and digested with trypsin (Promega, Madison, WI). The resulting peptides were separated by strong cation exchange chromatography, and affinity purified by avidin cartridges following the manufacturer’s protocol (Applied Biosystems), through which the cysteine (Cys)-containing peptides were enriched. The Cys-containing peptides were then subjected to LC-MS/MS using an LCQ-DECA-XP ion-trap mass spectrometer.

### LC-MS/MS analysis and protein identification

Tryptic peptides from each of the gel slices were analyzed using an LTQ linear ion trap mass spectrometer (Thermo Finnigan, San Jose, CA), as described previously [27]. The solvent gradient of HPLC was linear from 100% solvent A (5% acetonitrile, 0.4% acetic acid, and 0.005% heptafluorobutyric acid) to 80% solvent B (100% acetonitrile, 0.4% acetic acid, and 0.005% heptafluorobutyric acid) for 78 minutes, with a 20-65- minute acquisition. For the ICAT samples, each full MS scan was followed by three MS/MS scans of the most intense ion with data-dependent selection using the dynamic exclusion option. Otherwise, each full MS scan was followed by five MS/MS scans. Mass spectrometry raw files were converted to .dat files using XCalibur software (Version 1.4 SR1). Dat files were then converted to mzXML using the conversion software dat2xml from the Institute for Systems Biology, Seattle, Washington. All mzXML files were searched against a local copy of the non-redundant mouse protein database (56,709 entries, November 30, 2014 release version) from the NCI, National Institutes of Health, Advanced Biomedical Computing Center using SEQUEST-PVM Version 27 [28]. Peak lists were generated automatically without smoothing and deisotoping, and charge states were assigned based on the MS and MS/MS scans [28]. SEQUEST search parameters were as follows: trypsin digestion; no filtering thresholds; mass tolerance of 3.0 Da for precursor ions and 0.0 for fragment ion tolerance; full tryptic constraint allowing one missed cleavage; differential modification of +16 allowed for methionine; static modification set to +227.13 for ICAT-labeled cysteine and +9 for heavy ICAT-labeled cysteine residues, with a maximum of 4 modified AAs per peptide. XPRESS was used to quantify ICAT peptides [29]. Briefly, XPRESS isolates light and heavy peptide elution profiles, determines the area of each peak and calculates the abundance ratio based on the areas. The list of matched light/heavy peptides was filtered by (a) presence of cysteine; (b) XCorr and dCn scores, as described below; and (c) heavy and light pairs exhibiting closely eluting peaks as determined by scan number. Search results were processed using INTERACT [29] and filtered using the following criteria: XCorr cutoff values of 1.9, 2.2 and 3.7 for 1+, 2+ and 3+ peptides, respectively; deltaCn cutoff value of 0.1; for ICAT data, partially isotope-labeled peptides were excluded, and matched light/heavy peptides were further filtered by (a) presence of cysteine and (b) heavy and light chain pairs exhibiting closely eluting peaks as determined by scan number. Three ICAT data sets, with identified protein numbers of 963, 236, and 190 were combined to form the final ICAT count of 1034. The largest data set (963) was re-searched against a combined forward/reverse mouse protein database (November 30, 2014 release; 56,709 fwd entries; 56,709 rvs entries added) to estimate the false discovery rate (FDR) [30]. FDR estimation verified a protein FDR < 1% associated with the filtering criteria used. Peptides identified in the original search were re-searched against the Release 2013_08 Uniprot Knowledgebase (fasta format, 50821 entries) to update protein ids and obtain amino acid coverage. Where peptides map to multiple proteins or to multiple isoforms, that redundancy is noted in the peptide lists under the column labeled '#DupProt' in ProteomicData.xlsx.

### Plasma membrane preparation

Plasma membranes from bone marrow derived macrophages (BMDMs) and B cells of gp96 KO and WT littermates were isolated using plasma membrane protein extraction kit according to manufacturer’s protocol (BioVision, Mountain View, CA). The plasma membrane fraction was dissolved in 0.5% Triton X-100 in PBS, and plasma membrane protein concentration was determined by Bradford assay.

### Antibodies and western blotting

Antibodies to CD180, WDR1 and CAPZB were from Abcam; to GANAB were from Proteintech; to gp96 (Clone 9G10, SPA-851) were from Enzo Life Science; to β-actin was from Sigma. Blotting used 30 μg of protein lysate. Essentially all procedures were performed as described previously [31, 32].

### Immunoprecipitation

PreB leukemia cells were transduced with HA-tagged gp96 retrovirus and selected with 5 μg/ml blasticidin to generate a stable gp96 overexpressing cell line. Cells were then lysed on ice in radioimmunoprecipitation assay (RIPA) lysis buffer plus protease inhibitor cocktail (Sigma-Aldrich). Five hundred μg of lysate was pre-incubated with 2 μg of normal IgG antibody-conjugated protein G beads for 1 hour at 4°C to eliminate non-specific binding on antibody and protein G beads. After spinning, the supernatant were transferred to a new tube that contained 2 μg of anti-HA antibody (Sigma-Aldrich, clone: HA-7)-conjugated protein G beads and incubated for another 3 hours at 4°C. After washing, proteins were eluted with 5X SDS loading sample buffer from the beads and loaded for a SDS PAGE, followed by protein identification by LC-MS/MS. The acquired MS/MS data were searched against the NCBInr mouse database (NCBInr 2015.03) with Mascot (v2.3.2, Matrix Science) using Mascot Distiller (2.3.2.0) as the data input filter to generate peak lists. Search parameters were set as follows: enzyme, trypsin; precursor ion mass tolerance, 10 ppm; fragment ion mass tolerance, 0.7 Da; maximum missed cleavages allowed 2; carbamidomethyl of cysteine residues for fixed modification; oxidation of methionine for variable modification. The criteria used to filter results included 1% false positive threshold and expect value of less than 0.05 for significant peptide matches. The expect score was calculated using the homology threshold or the significance threshold as per a standard Mascot protein family report. Proteins represented by less than two peptides, or present in less than two LC-MS/MS runs were excluded from further analysis. Proteins identified by a subset of peptides from other proteins were filtered out from the results, and those matching the same set of peptides were grouped together into clusters.

### Gene ontology (GO) analysis

GO analysis was conducted using the Cytoscape Plugin (Bingo 2.44) tool to calculate enrichment of gene ontology [33], Analysis was done with default parameters and results corrected for multiple testing by the method of Benjamini and Hochberg [34].

## Results

### Comparative protein profiling from WT and KO gp96 BMDM by ICAT

**Fig 1:**
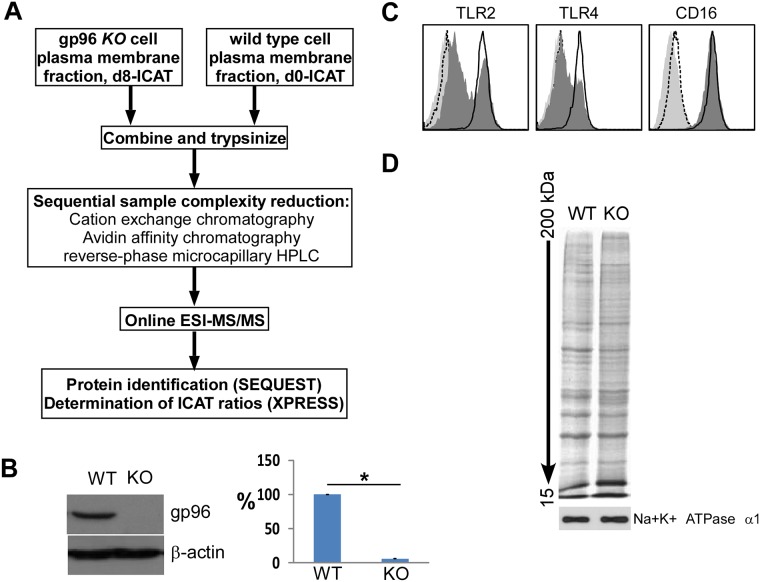
Proteomic analysis of plasma membrane proteins of macrophages whose expression is dependent on gp96. A) Schematic of ICAT-based method. B) IB of gp96 andβ–actin from WT and KO Mø. Quantitated data were shown in the bar graph. *p<0.01. Data represent three western blot results. Error bars denote s.e.m. C) Flow cytometric analysis of cell surface TLRs and CD16 (WT: open; KO: filled dark). The dashed and light histograms represent isotype stains. D) SDS-PAGE and silver staining of membrane proteins. The plasma membrane protein, Na^+^K^+^ATPase α1, was immunoblotted as a loading control.

We focused on the quantification and identification of plasma membrane (PM) proteins from WT and gp96 KO cells, as we predict that proteins requiring the chaperoning function of gp96 for cell surface transport must have reduced expression level in gp96 KO cells. We used two approaches. The first approach was to phenotypically profile surface proteins using flow cytometry. We found that KO and WT cells expressed the same levels of cell surface MHC II, CD14, CD16/32, CD43, CD44, CD80 and CD86 on macrophages (Møs) [17], and CD5, CD23, CXCR4, CCR7 and CD62L on B cells [18]. However, surface expression of TLR2, TLR4, α4, β2, β7 and αL was either reduced or absent from KO cells [18]. Our second approach was based on covalent tagging of the proteins with isotope-coded affinity tag (ICAT) reagents followed by proteolysis of the combined labeled protein samples, isolation, identification, and quantification of the tagged peptides by multidimensional chromatography, automated tandem mass spectrometry (MS) and computational analysis of the obtained data (Fig 1A) [29, 35]. This method allowed us to identify and determine the ratios of abundance of each protein from KO vs. WT cells. We derived KO Møs from bone marrow of *LsyM*^*Cre*^*Hsp90b1*^*flox/ko*^ mice which had significant reduction of gp96 (Fig 1B). Flow cytometry confirmed the reduction of cell surface TLR2 and TLR4 on the KO cells (Fig 1C). Same quantities of PM proteins were then isolated from WT and KO cells (Fig 1D). By ICAT method, a total of 1,034 proteins were isolated and sequenced, 55 of which were reduced (S1 Table), and 49 of which were increased in KO BMDMs (S2 Table). Table 1 lists selective proteins (bolded) with reduced expression in KO cells, based on the fulfillment of all of the following criteria: (1) they are known plasma membrane proteins, (2) 3 or more peptides from the same protein were identified, (3) SD of the KO/WT ratio of multiple peptides was less than half of the mean. This method is reliable as the reduction of all of these proteins in KO cells has been confirmed by flow cytometry. β1 integrin is not a gp96-client, indicated by the KO/WT ratio of 1.09 from a total of 12 identified β1-derived peptides, which was confirmed by flow cytometry in multiple cell types. Meanwhile, we found that cytoskeleton-related proteins, such as radixin, Mikaa0051 and Wd-repeat protein1, were also decreased in gp96 KO BMDMs (Table 1).

**Table 1:** A selected list of reduced PM proteins in gp96 K BMDM.

Protein description	Function	Heavy/Light ratio*
Exocyst complex component 8	Exocytosis	0.66
**Integrin alpha-4 precursor**	**Cell adhesion**	**0.61**
**Integrin alpha-l precursor**	**Cell adhesion**	**0.5**
**Integrin alpha-v precursor**	**Cell adhesion**	**0.69**
**Integrin beta-2 precursor**	**Cell adhesion**	**0.7**
**Integrin beta-5 precursor**	**Cell adhesion**	**0.7**
Lipoprotein receptor-related protein1 (CD91)	Lipid homeostasis	0.43
Multidrug resistance-associated protein 5	Anion transporter	0.49
Radixin	Cytoskeleton	0.7
Transforming protein n-ras	GTPase	0.69
Retinoic acid early inducible protein 1 beta	Cell death	0.59
**Toll-like receptor 2 precursor**	**Immune response**	**0.51**
**Toll-like receptor 4 precursor**	**Immune response**	**0.64**
**Toll-like receptor 13 precursor**	**Immune response**	**0.49**
Mkiaa0051 protein	Cytoskeleton	0.65
Polymeric immunoglobulin receptor 3	lgA and IgM receptor	0.63
Phospholipase c, gamma 2	Lipid degradation	0.64
Wd-repeat protein 1	Cytoskeleton	0.65

*Plasma membrane proteins were selected in the ratio of heavy reagent-labeled (KO)/light reagent-labeled (WT) ≤ 0.7

### Comparative and quantitative 2-D gel electrophoresis (2-DE), coupled with mass spectrometry, demonstrated the reduction of membrane proteins from gp96 KO BMDMs

**Fig 2:**
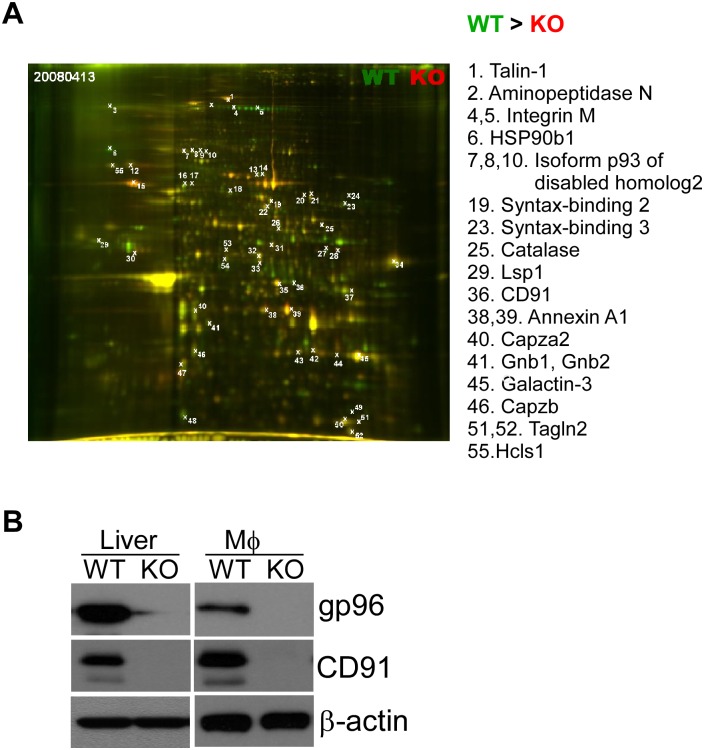
2-D electrophoresis identify CD91 as a novel interactor of gp96. A) Overlay of WT (green) and KO (red) plasma membrane proteins from macrophages after 2-D gel electrophoresis. 55 spots were analyzed, and reduced proteins in gp96 KO BMDMs were listed with the corresponding spot numbers. B) Proteins were extracted from both liver and Macrophage (Mø) of gp96 WT or KO mice. CD91, gp96 and β-actin were immunoblotted. β-actin was a loading control. Data are representative of three experiments.

To further probe the differences in PM proteins between WT and KO BMDMs, we performed 2-DE of WT and KO membrane proteins side by side. Proteins were detected by SPPRO Ruby staining. WT PM proteins were stained with Gy3 (Green), whereas KO PM proteins were labeled with Cy5 (Red). WT and KO gels were overlaid, and then 55 spots were picked for sequencing (Fig 2A). We found that 17 of the proteins were reduced, and 33 of the proteins were increased in gp96 KO cells (S3 Table), compared to WT cell levels (Fig 2A). 15 of these reduced proteins were potential new interactors of gp96, including Tln1 (spot #1), LSP1 (spot #29), CD91 (spot #36), annexin A1 (spot #38, 39), Capzb (spot #46) and others (Fig 2 and S3 Table). The reduction of CD91 in KO cells was particularly intriguing since CD91 has been implicated as a gp96 receptor [36–38] but was questioned by others [39]. By immunoblot, we found that CD91 was indeed reduced in both gp96 KO liver and Møs (Fig 2B).

### Mapping gp96 network in B cells

**Fig 3:**
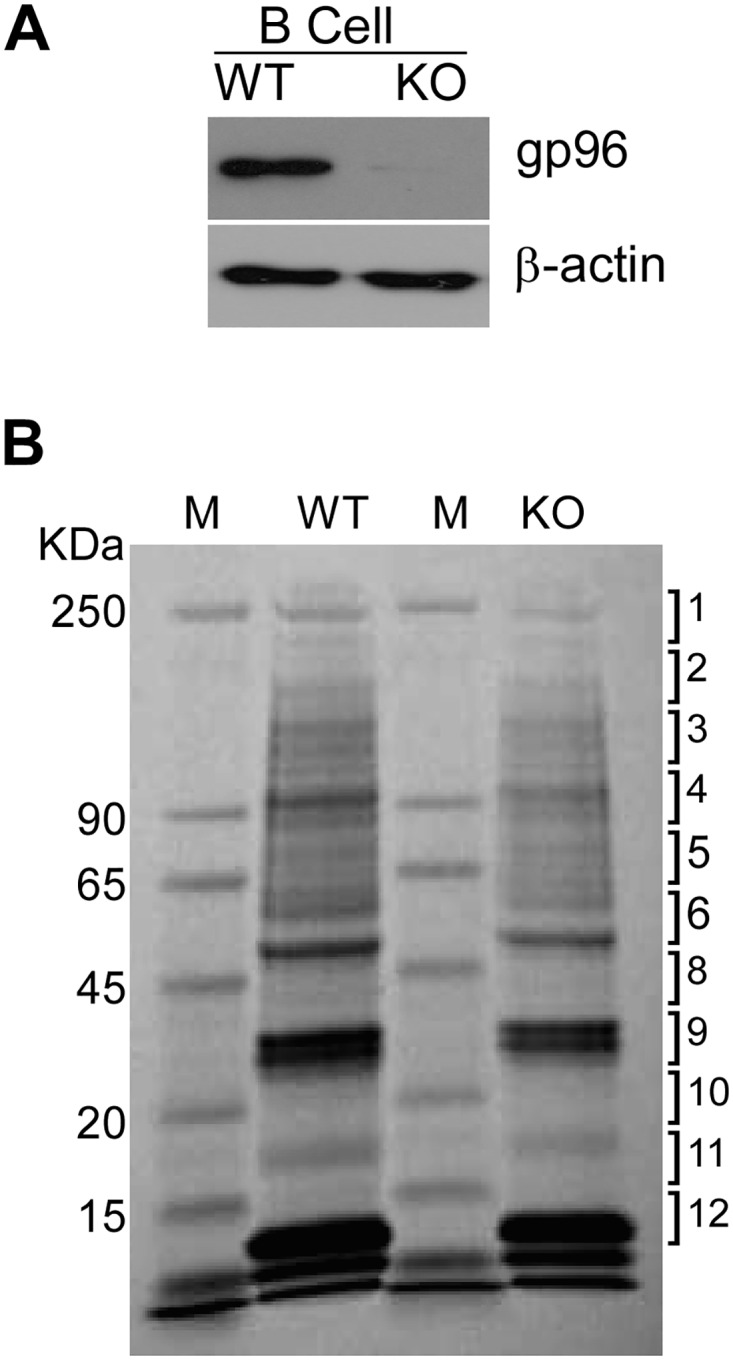
PM preparation from WT and gp96 KO B cells for protein profiling. A) Western blot of gp96 and β-action from gp96 WT and KO B cells. B) 50 μg of protein lysate was separated on SDS PAGE. The gel was stained by coomassie blue, and then cut into 12 pieces for protein identification.

Gp96 has been shown to optimize B cell function via chaperoning TLRs and integrins [18–20], suggesting that gp96 plays important roles in B cell biology. To further define the gp96 interactome in B cells, we took advantage of the B cell-specific gp96 null mice [18]. We purified B cells using CD19 microbeads and confirmed that gp96 level was significantly reduced in KO B cells, compared to WT B cells (Fig 3A). The equal amount of proteins isolated from the plasma membrane of both WT and KO B cells was separated by SDS-PAGE, and then the gel was cut into ten pieces across the molecular weight spectrum for protein identification by using MS spectrometry (Fig 3B). Comparing to WT B cells, a total of 444 proteins were reduced (S4 Table), and 23 proteins were increased in KO B cells (S5 Table). Since gp96 is crucial for the folding of proteins in the secretory pathway, we collected the plasma membrane proteins that were dramatically reduced in KO B cells (Table 2). Class II MHC and integrin β2 were confirmed, which were previously identified as gp96 clients [40, 41]. Lymphocyte-specific protein 1 (LPS1), a specific marker of leucocyte, is found in the wide range of lymphomas and leukemias, particularly of B cell origin [42]. This protein was also reduced significantly in gp96 KO B cells (Table 2). Once again, cytoskeleton proteins (rho-associated protein kinase 2, radixin and wd-repeat protein) were found to be decreased in gp96 KO B cells, comparing with WT B cell levels.

**Table 2:** A partial list of reduced PM proteins in in gp96 KO B cells.

Protein description	Function	KO/WT ratio*
Beta-a-microglobulin precursor	Peptide antigens presentation	0.21
Anion exchange protein 2	Anion exchange	0.50
CD180 antigen precursor	Immune response	0.25
Calpain-1 catalytic subunit	Proteolysis	0.33
Platelet glycoprotein 4 (CD36)	Cell adhesion	0.50
ADP-ribosyl cyclase 1 (cd38)	Second Messenger	0.38
CD97 antigen precurso(CD97)	leukocyte migration	0.43
Chloride intracellular channel protein 4	Chloride ion transporter	0.43
Complement receptor type 2 precursor	B lymphocyte activation	0.33
Alpha-enolase	Glycolysis	0.25
Immunoglobulin gamma Fc region receptor	Immune response	0.33
Growth factor receptor-bound protein 14	Inhibition of Insulin pathway	0.50
Solute carrier family 2	Glucose transporter	0.50
H-2 class I histocompatibility antigen	Immune response	0.29
Intercellular adhesion molecule 1 precursor	Cell adhesion	0.50
cAMP-dependent protein kinase	Protein phosphorylation	0.25
Lymphocyte-specific protein 1 (LSP1)	Neutrophil activation	0.40
Radixin	Cytoskeleton organization	0.30
Rho-associated protein kinase 2	Cytoskeleton formation	0.20
Synaptosomal-associated protein 23	Vehicle trafficking	0.38
Protein EFR3 homolog A OS	No information	0.50
Talin-1	Cytoskeleton	0.33
Adaptin ear-binding coat-associated protein 2	Endocytosis	0.33
Pleckstrin homology domain-containing family O member 1	Cytoskeleton	0.33
Integrin beta-1 precursor	Cell adhesion	0.50
Integrin beta-2 precursor	Cell adhesion	0.24
Leukocyte surface antigen CD47 precursor	Cell adhesion, T cell activation	0.44
Carboxypeptidase M precursor	Control of peptide hormone and growth factor activity	0.14
Chitinase-3-like protein 3 precursor	Inflammation and allergy	0.33

*Plasma membrane proteins were selected in KO/WT ratio ≤ 0.5

### gp96 interaction network in preB leukemia cells

**Fig 4:**
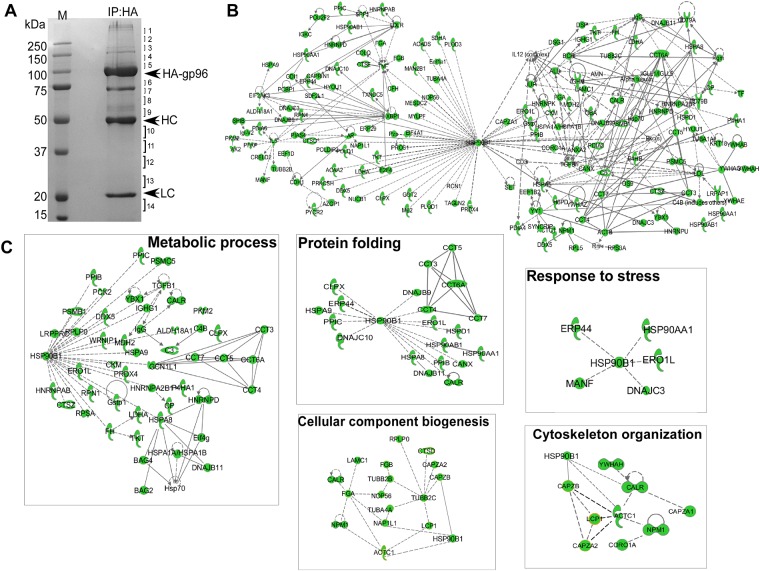
Mapping the gp96 interaction network. A) PreB cells infected with HA-gp96 retrovirus were selected with 5 μg/ml blasticidin for 5 days. HA-gp96 from the total cell lysates was immunoprecipitated with HA antibody. The eluate was resolved on SDS—PAGE and stained with Coomassie blue. The gel was cut into 14 pieces as indicated, trypsin-digested and sequenced by mass spectrometry. HC and LC represent Ig heavy chain and light chain, respectively. B) 130 putative gp96 interactors identified in PreB IP dataset were mapped into a network based on the literature available in the IPA database. Each direct or indirect interaction is supported by at least one piece of evidence from the scientific literature. Solid line showing direct interaction; dotted lines showing indirect or no interaction. C) Subnetwork of GO processes contained within the gp96 interaction network: metabolic process, protein folding, response to stress, cytoskeleton organization and cellular component biogenesis.

To further expose the potential interactors of gp96 in cancer cells, we tagged gp96 with hemagglutinin (HA) epitope from the influenza virus and carried out immunoprecipitation using the anti-HA antibody. We pulled down gp96-associated proteins from gp96-HA overexpressing preB leukemia cells, followed by SDS-PAGE and protein identification by LC-MS/MS (Fig 4A). A total of 201 gp96-associated proteins were selected from 801 clean entries with expectation value smaller than 0.05 (S6 Table) (See detail in “Materials and Method” section). Using the Ingenuity Pathway Analysis (IPA) tool, 130 of them were found in the IPA database. The overall interactive character of gp96 network was created and evaluated by statistical analysis (Fig 4B). Most of these proteins did not show direct interaction with gp96 due to absence of literature support for client proteins and cofactors in the IPA database. However, these results suggested a number of potential interaction proteins and cofactors that are involved in many important biological processes, including MesD, an ER chaperone for Wnt co-receptor LRP5/6. We have previously shown that MesD functions as a co-chaperone of gp96 to fold LRP6 [43]. The connectivity of gp96 targets was further exploited by extracting from the global gp96 network and assigned to specific GO category subnetworks including metabolic process, protein folding, response to stress, cytoskeleton organization and cellular component biogenesis (Fig 4C).

### Overview of gp96 interactome

**Fig 5:**
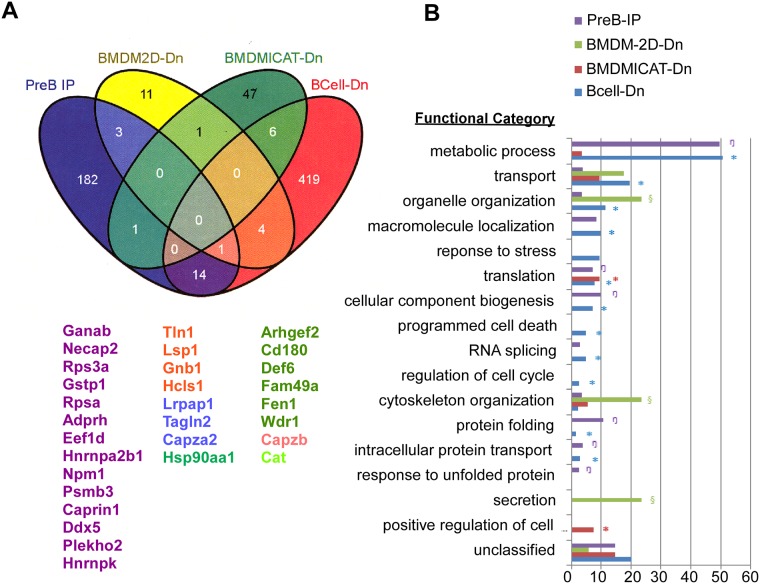
Overview of the gp96 proteomic data. A) A list of selected gp96 interactors discovered by multiple strategies in this study (see text for more details). B) Functional distribution of gp96-associated proteins using the DAVID database compared to the whole mouse genome. Colored symbols refer to statistical enrichment of certain types of genes in a given mapping method as compared to the whole mouse genome. All colored symbols indicate p<0.05.

The combined four different methods resulted in the identification of a total of 589 proteins that were interaction proteins of gp96. Collectively, these data provide a comprehensive framework for understanding the relationships among the specific biochemical pathways linked to gp96. Fig 5A shows a list of selective gp96 interactors. We do not expect to see significant overlap of interactors discovered due to difference in methodology (IP vs. ICAT) and cells (B cells vs. macrophages). The proteins identified in preB cells (preB IP) using the IP method are more likely to represent direct interaction with gp96, whereas the proteins identified in B cells (BCell-Dn) using the LC-MS/MS method are the largest dataset that shows 444 of proteins reduced in gp96 KO B cells. These two datasets shared 14 proteins in common, including Ganab, Necap2, Rps3a, Gstp1, Rpsa, Adprh, Eef1d, Hnrnpa2b1, Npm1, Psmb3, Caprin1, dDx5, Plekho2 and Hnrnpk). Six of the proteins (Arhgef2, CD180, Def6, Fam49d, Fen1 and Wdr1) that shown in the BCell-Dn dataset also presented in BMDMICAT-Dn dataset. The BCell-dn dataset also share 5 proteins (Tln1, Lsp1, Gnb1, Hcls1 and CapzB) with the BMDM2D-Dn dataset. Overall, we expanded the potential gp96 interactome by probing multiple cell types with multiple complimentary approaches. The expected non-overlapping nature of many of the proteins indicates the limitation of each method on its own, and points out the dynamic and cell type-specific interaction between gp96 and its associated proteins.

The interaction datasets were subsorted into functional categories using the Database for Annotation, Visualization and Integrated Discovery (DAVID) [44, 45]. Statistical enrichment (p<0.01) within these categories was assessed. While the proteins obtained in each of the four mapping methods were distributed across all major functional categories, the gp96 interactors were specifically enriched in metabolic process, transport, macromolecule localization, translation, cellular component biogenesis, cytoskeleton organization and protein folding (Fig 4B).

### Confirmation of reduced proteins in gp96 KO cells or tissues

**Fig 6:**
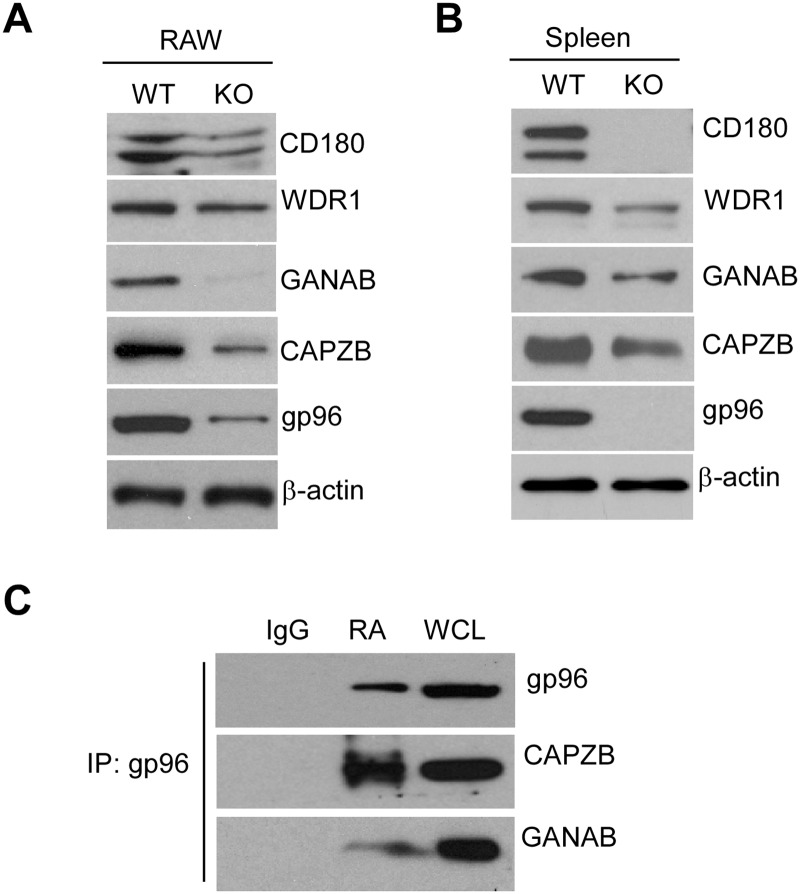
Proteins reduced in gp96 KO cells or tissues. A and B) Protein levels of CD180, WDR1, GANAB, CAPZB and gp96 in WT and KO gp96 RAW cells (A) or spleen tissues (B) were measured by Western blot. β-actin was a loading control. C) Anti-gp96 precipitates were resolved by SDS PAGE, and GANAB, CAPZB and gp96 were immunoblotted. IgG is an isotype control for Immunoprecipitation. WCL: whole cell lysate input.

We have used four independent datasets by using physical (preB IP) and genetic methods (BCell-Dn, BMDMICAT-Dn and BMDM2D-Dn) to map gp96 interactome. To further validate that these proteins are regulated by gp96, we performed western blot and immunoprecipitation studies to determine if they can be associated with gp96. We knocked down (KD) gp96 in RAW264.7 macrophage (RAW) using an shRNA strategy, and found that the protein levels of CD180, WDR1, GANAB and CAPZB were concordantly decreased in gp96 KD RAW cells, comparing with levels in WT RAW cells (Fig 6A). This reduction was also observed in the whole spleen lysates of gp96 KO mice (Fig 6B). Furthermore, we found that GANAB and CAPZB could be co-immunoprecipitated with gp96, suggesting that they are newly discovered gp96 interactors (Fig 6C).

## Discussion

gp96 is a ubiquitously expressed and evolutionarily conserved ER-resident molecular chaperone belonging to the HSP90 family [11]. Genetic and biochemical approaches have demonstrated the pivotal role of gp96 in folding and maturation of a rather select group of clientele to date, namely integrins and TLRs [17, 19]. Additionally, as a canonical member of HSP family, gp96 is known to participate in variety of molecular and biochemical processes in the cellular response to stress, the unfolded protein response (UPR), and the interplay between gp96 and OS9 in the ER-degradation pathway [46]. Thus, the functional importance of a molecule such as gp96 in the ER is two-fold: 1) it participates in the folding/maturation of two very important classes of immunologically relevant molecules, and 2) serves a second perhaps more general house-keeping role in ER homeostasis.

We have performed four complementary biochemical and genetic methods to map the gp96 interactome in B cells and macrophages. The combination of four datasets, one based on physical interaction and three based on genetic comparison, provides for a unique perspective on the varied physiological roles of gp96. We believe that the use of multiple methodologies is necessary due to several considerations. First, 2D-PAGE analysis was accurate but not sensitive comparing with other methodologies (i.e., only 55 differently expressed proteins were identified). Second, whereas ICAT strategy focused on identifying protein with reduced expression level in gp96 KO cells, gp96 pull-down study required strong protein-protein interaction. Third, due to difference in protein expression level and pattern, multiple cell lines (BMDM, B cell and preB cell) are needed to map the complete gp96 interactome. Due to the last consideration, more future work needs to be done to identify gp96 interactors in other cell types during both physiological and pathological conditions. Nevertheless, our data collectively indicate that gp96 plays a central role in protein folding, stress response, metabolic process, cytoskeleton structure, transport and cellular component biogenesis.

Cells exercise control over the quality of secretory proteins both by promoting their proper folding and by detection and disposal of misfolded molecules. Gp96 is critical for folding the secretory proteins. From our three genetics proteomics approaches, we identified a number of reduced hits in KO cell line, which was much more than increased hits. This could be due to 1) Interaction proteins could not be transported to cell surface because they are immature in the absence of gp96; 2) Interactors underwent ER-associated degradation and caused reduction of total expression level when gp96 was deleted. Obviously, protein reduction often triggers compensatory increase of other proteins, such as upstream molecules. The increased proteins we identified might be important components in gp96-regulated secretory pathways. Further studies are needed to verify these regulators.

201 proteins were found to interact with gp96 in PreB IP dataset (S6 Table). These proteins could include client proteins, cochaperones and co-factors. We previously demonstrated that CNPY3 is a cochaperone of gp96, which specifically folds TLRs, but not integrins [21], suggesting that gp96 might require different cochaperones or cofactors to fold specific client proteins. Using immunoprecipitation method, we found that, besides CNPY3, the other two members of CNPY family, CNPY2 and CNPY4, also associated with gp96 (Fig 4B). Our unpublished data showed that shRNA to CNPY4 led to reduction in surface expression of integrins although CNPY4 had been previously shown, as a TLR4-associated protein, to regulate surface expression of TLR4 [47]. Other potential cochaperones or cofactors could be Hspa5, Dnajc3, Dnajc10, Ero1I, Erp44, Hspd1 and Npm1 (S6 Table). All of these proteins have ER localization, binding to gp96, response to stress or unfolded proteins, and are related to various cellular process. This raised a possibility that gp96 might chaperone client proteins together with different specific cochaperones or cofactors much like its cytosolic counterpart, HSP90, which has a number of defined cochaperones and cofactors [48].

We and others have shown that gp96 chaperones all of TLRs except for TLR3 [18, 19, 21]. TLRs share a common leucine-rich repeat (LRR) motif, which is a protein structural motif that forms an α/β horseshoe fold [49]. LRR motifs are frequently involved in the formation of protein-protein interactions [50], and have been identified in a large number of functionally unrelated proteins. Thus, gp96 might interact with TLRs through binding to the LRR motifs. Recently, we demonstrated that gp96 binds and folds three other LRR motif-containing proteins, gpIX, gpV and GARP [41, 51]. Furthermore, the best known LRR proteins, ribonuclease inhibitor (Rnh1) and Leucine-rich repeat-containing protein 59 (LRRC59), were also shown in the BCell-Dn dataset (S4 Table). These suggested that gp96 might chaperone a specific category of proteins that contain the LRR motif.

Consistent with the importance of gp96 protein in biology, loss of gp96 protein during development is embryonic lethal [52]. Our data showed that gp96 indeed regulates development of multiple organs, including brain, heart, lung, liver, intestine and muscle, through modification of multiple different proteins. Nine of proteins, including CD91, Mtx1, Itgb1, Mapk14, Gatad2a, Psmd12, Sp1, Crry, and Hba-a2, were decreased in expression in gp96 KO B cells or BMDMs. All of these proteins were previously shown to regulate embryonic development [53–59], suggesting that gp96 could potentially regulate embryonic development through the folding of these proteins. Interestingly, CD91, a receptor for extracellular gp96 [36], were also controlled by gp96 (Fig 2B, Table 1), indicating that gp96 might perform self-control by adjusting the level of its receptor upon stimulation. This mechanism may be important for quality and safety control of gp96 action inside cells.

Previous studies demonstrated that gp96 is presented on the cell surface [60], in the Golgi apparatus [61], nucleus [62] and extracellular milieu [63], although it is mainly expressed in the ER. Gp96 can interact with apolipoprotein and BSDL outside the ER [64, 65]. We found here that the level of cytoskeleton-related proteins, WDR1 and CAPZB, were reduced in gp96 KO RAW macrophages and spleen tissue (Fig 6A and 6B). Moreover, CAPZB bound to gp96 (Fig 6C). Some other cytoskeleton proteins, including Actc1, Capza1, Npm1, Calr and Coro1a (S6 Table), Radixin and Rock2 (Table 2) were also reduced in gp96 KO cells, suggesting that gp96 may play an important role in cytoskeleton organization outside the ER.

The function of gp96 suggests that it may also play important roles in cancer biology, such as breast cancer [66], lung cancer [67], colon cancer [68], and esophageal squamous cell carcinoma [69]. Higher gp96 level is usually correlated with higher pathological grade and worse clinical outcome. However, it remains unknown which client proteins of gp96 are involved with cancer. Recently, we demonstrated that GARP, a gp96 client, promotes oncogenesis and immune tolerance through regulation of T regulatory cells in breast cancer [70]. Our PreB leukemia dataset showed that a number of molecules, as interaction proteins of gp96, have been implicated in oncogenesis. For example, NPM1, a DNA and unfolding protein binding protein, has been shown to regulate ARF/p53 pathway and correlate with acute myeloid leukemia and liver cancer [71, 72]. Tubulin α4a (TUBA4A) is involved in a variety of tumors. Many modulators of tubulin are currently in clinical trials for cancer therapy. Other molecules, such as CP, CERLD2, CTSD, HSPA5, HTR3A and TUBB2C, are also frequently up-regulated in cancer. Thus, gp96 plays important roles in cancer biology, probably through modulation of these cancer-related proteins. Gp96 therefore will be an attractive drug target for cancer therapy.

In conclusion, our multiple complimentary strategies provided a comprehensive view of gp96 network that includes its potential client proteins and other interactors. Further studies are needed to verify these potential clients, cochaperones and co-factors that were hinted here in this investigation. Through chaperoning its clientele, gp96 appears to be strategically positioned in the secretory pathway to integrate innate immunity and organ development. This current study sheds light on understanding the physiological and pathological roles of gp96.

## Supporting Information

S1 Table Reduced PM proteins in gp96 KO BMDMs. 1034 genes were obtained from BMDM ICAT protein profiling, and then selected with a heavy (KO)/light (WT) ratio less than 0.7. 55 selected genes were input into search engine for GO annotation enrichment.
(PDF)

S2 Table Increased PM proteins in gp96 KO BMDMs. 1034 genes were obtained from BMDM ICAT protein profiling, and then selected with a heavy (KO)/light (WT) ratio more than 1.5. 49 selected genes were input into search engine for GO annotation enrichment.
(PDF)

S3 Table Comparison of PM protein levels of gp96 WT and KO BMDMs. 2-DE imagine quantitation and MS/MS analysis was carried out as described in Methods. 55 spots were analyzed, and shown in blue with protein level of WT BMDMs greater than that of KO BMDMs (WT>KO), otherwise, shown in red with KO>WT.
(PDF)

S4 Table The list of reduced proteins in gp96 KO B cells. 443 of proteins down-regulated in gp96 KO B cells were selected from 1425 probes based on MS/MS results. The selection cutoff was WT/KO larger or equal than 2. Enrichment GO annotation was done after.
(PDF)

S5 Table Increased proteins in gp96 KO B cells. 23 of proteins up-regulated in gp96 KO B cells were selected from 1425 probes based on MS/MS results. The selection cutoff was WT/KO less or equal than 0.5.
(PDF)

S6 Table gp96-associated proteins in preB cells. 811 clean entries were detected from Immunoprecipitation method. A total of 201 genes were selected from those 811 genes with expectation value small than 0.05.
(PDF)
