# Supplementary material for: Mapping the Interactome of a Major Mammalian Endoplasmic Reticulum Heat Shock Protein 90
Source: PLoS One. 2017 Jan 5;12(1):e0169260. doi: 10.1371/journal.pone.0169260 (PMC5215799; doi:10.1371/journal.pone.0169260)
Supplement: S1 Table — 1034 genes were obtained from BMDM ICAT protein profiling, and then selected with a heavy (KO)/light (WT) ratio less than 0.7. 55 selected genes were input into search engine for GO annotation enrichment. (PDF) [file pone.0169260.s001.pdf]

S1 Table: Reduced PM proteins in gp96 KO BMDMs

Accession	Gene Symbol	MW	PI	Hydro	KO/WT ratio	StdDev	peptide count	UniqPep Count	aa Coverage
Q60875	Arhgef2	68585	9.2	-0.1317952	0.67	0.01	2	1	1.50%
Q9QZB7	Actr10	46222	7.54	0.090048	0.52	0.16	2	1	3.40%
Q9CTG6	Atp13a2	126614	8.49	0.1632678	0.56	0	1	1	1.70%
P05555	Itgam	127481	6.87	0.0362099	0.91	0.14	30	5	8.10%
P24270	Cat	59634	7.72	-0.0671103	0.65	0.1	3	2	4.70%
Q62192	Cd180	74267	5.53	0.1115129	0.69	0.29	21	6	12.60%
Q6SJQ7	Cd300lf	30777	4.72	0.1085512	0.63	0.19	2	1	9.20%
P56391	Cox6b1	9940	8.97	-0.1471765	0.33	0	1	1	12.80%
Q80YE7	Dapk1	161442	6.43	0.0006449	0.69	0	1	1	1.20%
Q8C2K1	Def6	73437	6.1	-0.2378411	0.55	0	1	1	2.70%
Q6PAL8	Dennd5a	146653	6.25	-0.0112044	0.53	0	1	1	0.90%
P11835	Itgb2	85026	7.02	-0.0514656	0.81	0.14	69	9	15.70%
Q9QZD8	Slc25a10	31715	9.43	0.0916028	0.46	0	1	1	5.20%
Q3UH60	Dip2b	83516	7.26	0.0938423	0.63	0	1	2	2.20%
O08553	Dpysl2	62170	5.95	0.0343707	0.68	0	1	1	2.80%
Q8BFR5	Tufm	47222	7.65	0.0425517	0.67	0	1	1	2.70%
Q6PGF7	Exoc8	81035	5.29	-0.0654888	0.66	0	1	1	2.00%
Q7M759	Fam108b1	10468	5.02	0.0559341	0.60	0.17	2	1	5.59%
Q8BHZ0	Fam49a	37343	5.71	-0.0377709	0.61	0	1	1	6.80%
P39749	Fen1	42315	8.54	-0.1021429	0.48	0	1	1	6.90%
P97379	G3bp2	54088	5.44	-0.1870953	0.59	0.14	2	1	2.50%
Q8JZK9	Hmgcs1	57553	5.65	0.0063077	0.62	0	1	1	1.90%
Q91VB4	Hps3	113152	5.4	0.0698903	0.69	0	1	1	1.60%
P07901	Hsp90aa1	84657	4.93	-0.1453687	0.63	0	2	2	4.00%
Q00651	Itga4	115696	6.28	0.0395188	0.61	0.11	5	3	4.70%
P24063	Itgal	128344	5.75	0.0397507	0.50	0.01	3	1	1.50%
P43406	Itgav	115278	5.46	0.0360537	0.69	0.1	4	3	3.60%
Q91ZX7	Lrp1	504742	5.14	-0.0668135	0.43	0.2	80	20	6.60%
A1L314	Mpeg1	73167	5.91	0.0667777	0.63	0.32	44	6	16.70%
Q9R1X5	Abcc5	161130	8.87	0.0638162	0.49	0.01	2	1	1.90%
Q8K2T1	Nmral1	34376	6.37	-0.0073139	0.60	0	1	1	5.20%
Q8CIH5	Plcg2	147592	6.34	-0.1078496	0.64	0.48	2	2	2.70%
Q99K51	Pls3	70742	5.42	-0.0064762	0.69	0	1	1	3.80%
Q8BJY1	Psmd5	55841	5.13	0.054175	0.65	0.08	2	1	3.20%
O08603	Raet1b	28625	9.18	0.0003952	0.59	0	1	1	3.60%
P08556	Nras	21199	5.01	-0.0346032	0.69	0	1	1	7.90%
P62717	Rpl18a	20732	10.72	-0.1981818	0.61	0	1	1	8.50%
P58283	Rnf216	83612	4.81	-0.0537568	0.33	0	1	1	1.60%
P62983	Rps27a	9404	9.86	-0.39175	0.63	0	2	1	8.30%
P62908	Rps3	29754	9.75	-0.148137	0.69	0	1	1	4.90%
Q64213	Sf1	70408	8.98	-0.0713936	0.60	0	1	1	1.80%
P07214	Sparc	34450	4.77	-0.0127815	0.65	0	1	1	4.00%
O70309	Itgb5	87909	5.81	-0.010401	0.71	0.05	3	1	2.10%
Q8BYC6	Taok3	37090	6.28	-0.0015124	0.50	0	1	1	0.90%
Q922F4	Tubb6	50090	4.8	0.0175839	0.62	0.14	5	1	3.80%
Q6R5N8	Tlr13	114443	8.85	0.0648638	0.49	0.22	9	2	2.30%
Q9QUN7	Tlr2	89449	6.19	0.0603572	0.51	0.03	3	1	1.70%
Q9QUK6	Tlr4	95519	6.12	0.1033175	0.64	0	1	1	1.60%

Q8BVW3	Trim14	51240	8.55	-0.0754305	0.47	0	1	1	3.00%
O88342	Wdr1	66407	6.11	0.0525083	0.65	0.62	6	3	10.60%
Q920I9	Wdr7	163389	6.42	0.0586771	0.50	0	1	1	0.90%
Q9JKF1	Iqgap1	191312	6.15	-0.0696981	0.65	0.11	41	6	4.90%
Q6NXL1	Sec24d	48661	7.94	0.0770507	0.46	0	1	1	1.60%
Q8BGF0	AW551984	85362	6.12	0.0444954	0.58	0	1	1	2.00%
Q8CGC6	Rbm28	56311	9.67	-0.2669798	0.48	0.15	3	1	2.50%

S1 Table : Reduced PM proteins in gp96 KO BMDMs. 1034 genes were obtained from BMDM ICAT protein profiling, and then selected with a heavy (KO)/light (WT) ratio less than 0.7. 55 selected genes were input into search engine for GO annotation enrichment.
