# Supplementary material for: Mapping the Interactome of a Major Mammalian Endoplasmic Reticulum Heat Shock Protein 90
Source: PLoS One. 2017 Jan 5;12(1):e0169260. doi: 10.1371/journal.pone.0169260 (PMC5215799; doi:10.1371/journal.pone.0169260)
Supplement: S2 Table — 1034 genes were obtained from BMDM ICAT protein profiling, and then selected with a heavy (KO)/light (WT) ratio more than 1.5. 49 selected genes were input into search engine for GO annotation enrichment. (PDF) [file pone.0169260.s002.pdf]

S2 Table: Increased PM proteins in gp96 KO BMDMs

Accession	Gene Symbol	MW	PI	Hydro	KO/WT	StdDev	peptide count	Unique Peptide	aa Coverage
P41233	Abca1	166966	5.33	0.0827334	1.72	0	2	2	1.10%
Q9DBE8	Alg2	47405	8.2	0.0433494	1.61	0	2	1	3.60%
Q6P1H6	Ankle2	80564	6.42	-0.0884457	1.69	0	2	1	1.20%
Q9JKW0	Arl6ip1	23437	9.38	0.1420197	1.61	0.09	8	2	4.90%
Q91YI4	Arrb2	46314	7.58	-0.0777317	2	0.38	2	1	8.30%
Q9EPE9	Atp13a1	132378	8.35	0.1109252	1.75	0	2	1	1.50%
Q02105	C1qc	25966	8.67	0.080813	1.79	0	2	1	4.90%
P14211	Calr	47995	4.33	-0.1623077	1.59	0.17	20	4	14.20%
O35350	Capn1	82106	5.62	-0.03108	2.86	0	2	1	3.20%
P23198	Cbx3	20855	5.13	-0.2287432	5	0	2	1	6.60%
Q8CIX9	Cd72	40493	6.36	-0.2247518	1.54	0	2	1	6.50%
Q99LL3	Chst12	49398	7.74	-0.0523389	4.55	0	2	1	2.40%
Q8BRT1	Clasp2	140739	8.84	-0.0839345	1.75	0	2	1	1.20%
Q6QLQ4	Clec7a	27564	6.83	-0.0234017	2.33	0	2	1	4.50%
P59108	Cpne2	61036	5.62	0.0573358	1.52	0.18	5	1	4.60%
O88456	Capns1	28463	5.41	0.106617	1.52	0	2	1	7.10%
P07141	Csf1	60649	5.1	-0.0772464	1.61	0.2	5	1	2.50%
P09581	Csf1r	109179	5.84	0.0407062	3.03	0	2	1	1.90%
Q8BG67	Efr3a	94146	6.98	-0.0078966	1.67	0	2	1	1.60%
P26151	Fcgr1	44888	5.73	0.0568812	1.72	0.07	4	1	6.70%
Q8R1W2	Gsg1	40613	5.82	0.126137	4.17	0	2	1	5.20%
P01897	H2-D1	40620	5.3	0.002873	1.92	0	3	3	13.80%
P06339	H2-T23	40875	5.91	-0.0172829	1.75	0.05	3	1	2.80%
P14428	H2-K1	41302	5.96	0.002873	1.96	0.11	2	3	13.80%
Q64345	Ifit3	47223	5.51	-0.1963771	1.92	0	2	1	4.00%
P20444	Prkca	76721	6.76	-0.0515052	1.69	0.02	2	1	2.10%
P48025	Syk	71376	7.92	-0.0344833	1.52	0.09	3	1	2.40%
Q8BSS9	Ppfia2	143234	5.79	-0.1994274	1.64	0	2	1	1.20%
P05533	Ly6a	14377	4.75	0.1890298	1.67	0.09	2	1	15.70%
Q9JMH9	Myo18a	230908	5.84	-0.1874886	1.52	0	2	1	0.50%
Q9QWR8	Naga	47235	6.02	0.0748915	1.64	0	2	1	2.90%
Q61194	Pik3c2a	187440	8.55	-0.00231	1.96	0	2	1	0.70%
P09103	P4hb	57144	4.79	-0.0068762	2	0	2	1	1.40%
Q922R8	Pdia6	48689	5.05	0.0281573	1.64	0.41	6	2	5.70%
Q8VD65	Pik3r4	72393	5.58	0.0142543	3.03	0	2	1	0.90%
P70182	Pip5k1a	60471	8.74	-0.020989	1.96	0	2	1	2.20%
O70161	Pip5k1c	72474	5.41	-0.0337368	1.59	0	2	1	1.80%
Q8BG07	Pld4	53390	8.41	0.069125	1.79	0.19	4	1	4.80%
Q9JKF6	Pvrl1	57064	6.06	-0.019301	1.82	0.03	2	1	2.50%
Q9QYF1	Rdh11	35148	9.1	0.1085759	1.54	0.12	3	1	3.80%
Q9WVM1	Racgap1	70158	8.78	-0.0777547	1.89	0.22	2	2	4.00%
Q6KAT6	Siglec1	33190	5.3	-0.0145763	2.04	0.14	35	9	10.90%
Q5FWI3	Tmem2	153815	7.88	-0.0008171	1.59	0	1	1	0.90%
Q9CR67	Tmem33	28031	9.72	0.1814171	1.54	0	1	1	6.90%
O35305	Tnfrsf11a	66621	4.94	-0.022896	1.59	0	1	1	2.90%
Q9D8Y7	Tnfaip8l2	20615	7.79	-0.0008696	1.96	0.11	2	1	7.60%
Q80X50	Ubap2l	107242	6.62	-0.0692008	1.85	0.05	4	1	1.60%
Q6P5E4	Ugcgl1	176553	5.38	0.007853	1.85	0	1	1	0.80%
Q8R5L3	Vps39	101693	6.53	0.01693	2	0.31	2	1	1.70%

S2 Table: Increased PM proteins in gp96 KO BMDMs. 1034 genes were obtained from BMDM ICAT protein profiling, and then selected with a heavy (KO)/light (WT) ratio more than 1.5. 49 selected genes were input into search engine for GO annotation enrichment.
