# Supplementary material for: Mapping the Interactome of a Major Mammalian Endoplasmic Reticulum Heat Shock Protein 90
Source: PLoS One. 2017 Jan 5;12(1):e0169260. doi: 10.1371/journal.pone.0169260 (PMC5215799; doi:10.1371/journal.pone.0169260)
Supplement: S3 Table — 2-DE imagine quantitation and MS/MS analysis was carried out as described in Methods. 55 spots were analyzed, and shown in blue with protein level of WT BMDMs greater than that of KO BMDMs (WT>KO), otherwise, shown in red with KO>WT. (PDF) [file pone.0169260.s003.pdf]

S3 Table: Comparison of PM protein levels of gp96 WT and KO BMDMs

	spot number	protein hit number	Accession	gene ID	gene name	score	peptide matches	number_matched (<0.05)	identify
WT>KO	1	1	IPI00465786	21894;	Tln1;	805	21	17	identification
WT>KO	2	1	IPI00465786	21894;	Tln1;	371	11	10	identification
WT>KO	3	1	IPI00319509	16790;	Anpep;	639	16	14	identification
WT>KO	4	1	IPI00120674	16409;	Itgam;	796	16	15	identification
WT>KO	5	1	IPI00120674	16409;	Itgam;	718	15	15	identification
WT>KO	6	1	IPI00129526	22027;	Hsp90b1;	574	21	15	identification
WT>KO	7	1	IPI00222828	13132;	Dab2;	337	8	5	identification
WT>KO	7	2	IPI00330695	227737;	9130404D14	248	12	5	identification
WT>KO	8	1	IPI00222828	13132;	Dab2;	375	12	8	identification
WT>KO	8	2	IPI00753430	227737;	9130404D14	65	1	1	candidate
KO>WT	9	1	IPI00751595	212032;	Hk3;	348	9	6	identification
WT>KO	10	1	IPI00222828	13132;	Dab2;	686	17	12	identification
KO>WT	11	1	IPI00229080	15516;	Hsp90ab1;	615	20	13	identification
KO>WT	11	1	IPI00330804	15519;	Hsp90aa1;	368	10	6	identification
KO>WT	12	1	IPI00228150	76614;	Immt;	253	10	8	identification
KO>WT	12	2	IPI00458392	69748;	Aldh16a1;	70	3	1	candidate
KO>WT	13	1	IPI00228150	76614;	Immt;	371	9	8	identification
KO>WT	14	1	IPI00228150	76614;	Immt;	146	5	5	identification
KO>WT	15	1	IPI00319992	14828;	Hspa5;	1553	23	23	identification
KO>WT	16	1	IPI00133903	15526;	Hspa9;	748	18	12	identification
KO>WT	17	1	IPI00133903	15526;	Hspa9;	685	16	11	identification
KO>WT	18	1	IPI00331182	14571;	Gpd2;	299	15	7	identification
WT>KO	19	1	IPI00133317	20911;	Stxbp2;	652	17	9	identification
KO>WT	20	1	IPI00137409	21881;	Tkt;	265	8	6	identification
KO>WT	21	1	IPI00137409	21881;	Tkt;	732	18	14	identification
KO>WT	22	1	IPI00318671	98878;	Ehd4;	611	18	13	identification
WT>KO	23	1	IPI00624098	20912;	Stxbp3a;	250	11	7	identification
KO>WT	24	1	IPI00129577	26926;	Aifm1;	175	6	5	identification
WT>KO	25	1	IPI00312058	12359;	Cat;	323	13	7	identification
KO>WT	26	1	IPI00221608	68653;	Samm50;	353	9	8	identification
KO>WT	27	1	IPI00130280	11946;	Atp5a1;	862	18	16	identification
KO>WT	28	1	IPI00130280	11946;	Atp5a1;	1000	20	18	identification
WT>KO	29	1	IPI00313672	16985;	Lsp1;	324	9	8	identification
KO>WT	30	1	IPI00468481	11947;	Atp5b;	912	19	15	identification
KO>WT	31	1	IPI00420385	52398;	Sept11;	315	10	7	identification
KO>WT	31	2	IPI00124372	56752;	Aldh9a1;	206	6	5	identification
KO>WT	31	2	IPI00115708	18718;	Pip5k2a;	87	1	1	candidate
KO>WT	32	1	IPI00462072	00044223;	Eno1	744	15	13	identification
KO>WT	32	2	IPI00112053	69178;	Snx5;	150	4	3	identification
KO>WT	33	1	IPI00318841	67160;	Eef1g;	241	10	10	identification
KO>WT	34	1	IPI00307837	13627;	Eef1a1;	416	10	7	identification
KO>WT	35	1	IPI00468924	229731;	Slc25a24;	258	14	7	identification
WT>KO	36	1	IPI00277930	12332;	Capg;	255	5	3	identification
WT>KO	36	2	IPI00469307	16976;	Lrpap1;	95	7	1	candidate
KO>WT	37	1	IPI00119138	67003;	Uqcrc2;	157	7	4	identification
WT>KO	38	1	IPI00230395	16952;	Anxa1;	654	17	11	identification
WT>KO	39	1	IPI00230395	16952;	Anxa1;	746	16	15	identification
WT>KO	40	1	IPI00111265	12343;	Capza2;	503	9	7	identification
WT>KO	41	1	IPI00162780	14693;	Gnb2;	137	5	3	identification
WT>KO	41	2	IPI00120716	14688;	Gnb1;	89	4	3	identification
KO>WT	42	1	IPI00122547	22334;	Vdac2;	401	6	6	identification

WT>KO	43	1	IPI00661414	76709;	Arpc2;	593	13	11	identification
WT>KO	44	1	IPI00230395	16952;	Anxa1;	259	6	5	identification
WT>KO	44	2	IPI00120051	16854;	Lgals3;	144	4	4	identification
WT>KO	45	1	IPI00131259	16854;	Lgals3;	343	7	7	identification
WT>KO	46	1	IPI00474883	12345;	Capzb;	278	10	9	identification
KO>WT	47	1	IPI00133440	18673;	Phb;	562	7	7	identification
KO>WT	48	1	IPI00230507	71679;	Atp5h;	483	7	7	identification
KO>WT	49	1	IPI00121788	18477;	Prdx1;	700	17	15	identification
KO>WT	50	1	IPI00121788	18477;	Prdx1;	571	17	16	identification
WT>KO	51	1	IPI00751340	21346;	Tagln2;	336	12	9	identification
WT>KO	52	1	IPI00751340	21346;	Tagln2;	353	12	11	identification
KO>WT	53	1	IPI00122565	14569;	Gdi2;	357	14	11	identification
KO>WT	54	1	IPI00128023	226646;	Ndufs2;	199	14	7	identification
KO>WT	54	2	IPI00221998	71881;	2310001A2(	61	4	1	candidate
WT>KO	55	1	IPI00117011	15163;	Hcls1;	605	15	12	identification

S3 Table: Comparison of PM protein levels of gp96 WT and KO BMDMs. 2-DE imagine quantitation and MS/MS analysis was carried out as described in Methods. 55 spots were analyzed, and shown in blue with protein level of WT BMDMs greater than that of KO BMDMs (WT>KO), otherwise, shown in red with KO>WT.
