# Supplementary material for: Mapping the Interactome of a Major Mammalian Endoplasmic Reticulum Heat Shock Protein 90
Source: PLoS One. 2017 Jan 5;12(1):e0169260. doi: 10.1371/journal.pone.0169260 (PMC5215799; doi:10.1371/journal.pone.0169260)
Supplement: S4 Table — 443 of proteins down-regulated in gp96 KO B cells were selected from 1425 probes based on MS/MS results. The selection cutoff was WT/KO larger or equal than 2. Enrichment GO annotation was done after. (PDF) [file pone.0169260.s004.pdf]

S4 Table: The list of reduced proteins in gp96 KO B cells

Accession	Gene Symbol	WT peptide	Normalized.SC.wt.	KO peptide	NormalizedSC.ko.	WT/KO ratio	MW	pI	Hydro	Unique Peptide	aa Coverage
P55194	Sh3bp1	3	0.00024	0	0	0	65285	5.49	-0.0370216	2	4.70%
P48193	Epb41	3	0.00024	0	0	0	95911	5.44	-0.1544987	1	1.60%
P56379	Mp68	3	0.00024	0	0	0	6698	10	0.10258622	1	13.80%
Q9CQ60	Pgls	11	0.00088	5	0.00052	2.2	27254	5.55	0.13389108	6	44.00%
Q61548	Snap91	2	0.00016	0	0.00000	0	91851	4.72	0.13849086	1	1.30%
P51912	Slc1a5	3	0.00024	0	0.00000	0	58483	8.14	0.24683544	1	3.60%
Q61102	Abcb7	3	0.00024	0	0.00000	0	82581	9.36	0.06853733	2	2.70%
Q8CBW3	Abi1	2	0.00016	0	0.00000	0	52288	7.16	-0.0602702	1	3.30%
Q8BYZ1	Abi3	4	0.00032	0	0.00000	0	39107	5.4	0.05029973	3	9.30%
Q9R0X4	Acot9	5	0.00040	2	0.00021	2.5	50560	8.74	-0.0203417	2	5.50%
Q8R5C5	Actr1b	2	0.00016	0	0.00000	0	42281	5.98	0.01388297	2	10.10%
Q8BK64	Ahsa1	2	0.00016	0	0.00000	0	38117	5.41	-0.0278107	2	11.80%
P45376	Akr1b1	2	0.00016	0	0.00000	0	35732	6.71	0.0365823	1	4.40%
O08663	Metap2	3	0.00024	0	0.00000	0	52922	5.56	-0.0978871	3	11.30%
O35639	Anxa3	3	0.00024	0	0.00000	0	36384	5.5	-0.0778019	2	5.30%
P28352	Apex1	18	0.00143	8	0.00083	2.25	35490	8.04	-0.0767508	6	27.40%
Q9WV32	Arpc1b	30	0.00239	11	0.00114	2.73	41064	8.68	0.02989247	7	22.60%
Q8CG76	Akr7a2	2	0.00016	0	0.00000	0.00	40612	8.36	-0.013188	2	7.10%
Q61210	Arhgef1	17	0.00135	8	0.00083	2.13	102805	5.43	-0.1080434	10	16.20%
Q8K4I3	Arhgef6	2	0.00016	0	0.00000	0.00	87051	5.66	-0.0577302	1	1.70%
P54923	Adprh	5	0.00040	0	0.00000	0.00	40068	5.46	0.06582873	3	17.40%
Q99MK8	Adrbk1	4	0.00032	0	0.00000	0.00	79639	6.89	-0.0674746	2	3.00%
O54984	Asna1	2	0.00016	0	0.00000	0.00	38823	4.81	0.07031608	1	2.90%
P01887	B2m	14	0.00111	3	0.00031	4.67	13779	8.55	0.03176469	1	7.60%
P13808	Slc4a2	2	0.00016	0	0.00000	0.00	136814	5.78	0.04562672	2	2.70%
O54962	Banf1	5	0.00040	2	0.00021	2.50	10103	5.79	-0.0723596	2	29.20%
Q07813	Bax	14	0.00111	3	0.00031	4.67	21395	4.86	0.0871354	3	24.00%
Q8K019	Bclaf1	2	0.00016	0	0.00000	0.00	106002	10	-0.4671148	1	1.00%
P70444	Bid	2	0.00016	0	0.00000	0.00	21952	4.71	-0.0458461	2	22.60%
Q9Z0H1	Wdr46	2	0.00016	0	0.00000	0.00	69048	9.79	-0.1121704	1	3.50%
P16277	Blk	9	0.00072	4	0.00042	2.25	56674	6.7	0.00444891	1	2.40%
Q91ZU6	Dst	2	0.00016	0	0.00000	0.00	833638	5.19	-0.1184446	2	0.30%
Q9WVA3	Bub3	14	0.00111	6	0.00062	2.33	36955	6.37	-0.0429448	4	19.60%
Q9JJ80	Rpf2	5	0.00040	2	0.00021	2.50	35364	10.05	-0.1773856	2	8.50%
Q62192	Cd180	4	0.00032	0	0.00000	0.00	74302	5.55	0.10971265	2	5.30%
Q61462	Cyba	11	0.00088	3	0.00031	3.67	20748	9.32	0.13708338	1	14.60%
Q61093	Cybb	9	0.00072	2	0.00021	4.50	65305	7.83	0.11470182	6	13.70%
O35744	Chi3l3	3	0.00024	0	0.00000	0.00	44458	5.42	0.06844224	2	8.50%
O35350	Capn1	3	0.00024	0	0.00000	0.00	82106	5.62	-0.03108	2	4.30%
P47757	Capzb	13	0.00103	6	0.00062	2.17	31345	5.47	-0.0946571	7	32.50%
P28293	Ctsg	2	0.00016	0	0.00000	0.00	29096	10.57	-0.0436399	1	4.20%
Q08857	Cd36	2	0.00016	0	0.00000	0.00	52698	8.6	0.0917161	1	2.50%
P56528	Cd38	8	0.00064	3	0.00031	2.67	34408	8.64	0.04868422	4	11.50%
Q9Z0M6	Cd97	7	0.00056	3	0.00031	2.33	90413	7.38	0.11443768	3	4.80%
Q9QYB1	Clic4	7	0.00056	3	0.00031	2.33	28729	5.44	-0.0310672	3	26.90%
Q08093	Cnn2	8	0.00064	3	0.00031	2.67	33156	7.53	-0.0200328	4	13.40%
Q9DAW9	Cnn3	6	0.00048	0	0.00000	0.00	36429	5.46	-0.0448182	4	22.10%
Q9DB96	Ngdn	2	0.00016	0	0.00000	0.00	35659	9.59	-0.2184762	1	4.40%
Q61387	Cox7a2l	3	0.00024	0	0.00000	0.00	12399	9.61	0.070991	2	40.50%
Q63829	Commd3	2	0.00016	0	0.00000	0.00	22037	5.36	-0.0495898	1	5.10%
O89079	Cope	3	0.00024	0	0.00000	0.00	34567	4.94	-0.0221429	2	10.70%
P19536	Cox5b	3	0.00024	0	0.00000	0.00	13813	8.69	-0.0172656	1	8.60%
P43024	Cox6a1	2	0.00016	0	0.00000	0.00	12352	9.97	-0.000991	1	16.20%

P19070	Cr2	3	0.00024	0	0.00000	0.00	112995	7.22	0.03824392	2	1.80%
Q8VBV7	Cops8	4	0.00032	0	0.00000	0.00	23256	5.09	0.07062201	3	20.10%
Q9DAT2	Mrgbp	2	0.00016	0	0.00000	0.00	23888	4.87	-0.0634404	1	6.00%
O88712	Ctbp1	2	0.00016	0	0.00000	0.00	47745	6.28	0.05133789	2	6.80%
P56395	Cyb5a	4	0.00032	0	0.00000	0.00	15241	4.96	-0.0293284	3	22.40%
Q9CXW3	Cacybp	4	0.00032	0	0.00000	0.00	26510	7.64	-0.206856	3	21.80%
Q80YW0	Cyth4	2	0.00016	0	0.00000	0.00	45285	6.24	-0.0852417	1	3.60%
Q9JIQ3	Diablo	2	0.00016	0	0.00000	0.00	26820	6.03	-0.0525738	1	8.40%
P54823	Ddx6	4	0.00032	0	0.00000	0.00	54192	8.85	-0.0106625	3	9.70%
Q9QX60	Dguok	5	0.00040	0	0.00000	0.00	32281	7.17	-0.0406498	3	21.70%
Q9CQA3	Sdhb	5	0.00040	0	0.00000	0.00	31814	8.96	-0.0615957	4	16.00%
Q8BZN6	Dock10	32	0.00255	11	0.00114	2.91	245758	6.63	-0.0512556	18	13.40%
Q8R1A4	Dock7	3	0.00024	0	0.00000	0.00	83399	6.14	0.0513378	2	10.40%
Q8C147	Dock8	15	0.00119	6	0.00062	2.50	238978	6.53	-0.0371524	19	11.70%
P52431	Pold1	2	0.00016	0	0.00000	0.00	123790	7.69	-0.0260362	1	1.30%
Q9QXB9	Drg2	2	0.00016	0	0.00000	0.00	40718	9.03	0.01659341	1	3.60%
P01882	IGHDM	14	0.00111	5	0.00052	2.80	32389	6.49	-5.84E-04	3	14.80%
Q9JHU4	Dync1h1	103	0.00820	31	0.00322	3.32	532045	6.03	-0.0352046	58	16.70%
O88487	Dync1i2	2	0.00016	0	0.00000	0.00	68394	5.16	-0.0887418	1	3.10%
P57776	Eef1d	11	0.00088	5	0.00052	2.20	31293	4.91	-0.1303914	4	18.10%
Q9CZR8	Tsfm	2	0.00016	0	0.00000	0.00	35334	6.62	0.02984567	1	8.30%
Q8BHL5	Elmo2	4	0.00032	0	0.00000	0.00	83887	5.65	-0.0224727	4	9.80%
P70290	Mpp1	3	0.00024	0	0.00000	0.00	52227	6.72	-0.0572103	3	12.40%
O08579	Emd	2	0.00016	0	0.00000	0.00	29436	4.85	-0.0555598	1	11.20%
P17182	Eno1	10	0.00080	4	0.00042	2.50	47141	6.37	0.02882489	8	33.90%
Q9D172	D10Jhu81e	5	0.00040	0	0.00000	0.00	28090	9	0.07026314	3	19.90%
Q99LC5	Etfa	3	0.00024	0	0.00000	0.00	35009	8.62	0.11264266	3	13.80%
P70429	Evl	21	0.00167	4	0.00042	5.25	44337	8.92	-0.1295169	9	33.10%
Q8BHZ0	Fam49a	9	0.00072	3	0.00031	3.00	37343	5.71	-0.0377709	1	4.00%
Q921M7	Fam49b	16	0.00127	7	0.00073	2.29	36776	5.76	-0.0379938	7	25.30%
P08101	Fcgr2	6	0.00048	2	0.00021	3.00	36695	6.24	0.01957575	5	21.20%
P39749	Fen1	5	0.00040	0	0.00000	0.00	42315	8.54	-0.1021429	3	7.10%
P14234	Fgr	2	0.00016	0	0.00000	0.00	58867	5.23	-0.0063443	2	3.70%
Q9R1C7	Prpf40a	5	0.00040	0	0.00000	0.00	108481	7.39	-0.2452149	3	2.10%
P97807	Fh	4	0.00032	0	0.00000	0.00	54357	9.12	0.07623274	2	5.10%
Q00612	G6pdx	3	0.00024	0	0.00000	0.00	59263	6.06	-0.0322136	3	9.70%
Q8BHN3	Ganab	2	0.00016	0	0.00000	0.00	106911	5.67	0.02565678	2	3.50%
P29387	Gnb4	5	0.00040	0	0.00000	0.00	37379	5.74	0.01873527	1	3.20%
P08752	Gnai2	21	0.00167	9	0.00093	2.33	40489	5.28	-0.0483098	6	21.10%
Q8BWF2	Gimap5	2	0.00016	0	0.00000	0.00	34653	6.76	-0.0454545	1	4.50%
Q9JLQ2	Git2	2	0.00016	0	0.00000	0.00	78766	7.64	-0.1588699	2	4.70%
P56400	Gp1bb	4	0.00032	0	0.00000	0.00	21763	10.51	0.12067965	1	8.30%
Q60631	Grb2	2	0.00016	0	0.00000	0.00	25238	5.89	-0.0795392	1	6.50%
Q9JLM9	Grb14	2	0.00016	0	0.00000	0.00	60573	8.69	-0.0557807	1	3.00%
P19157	Gstp1	3	0.00024	0	0.00000	0.00	23609	7.69	0.03733334	2	17.10%
P17809	Slc2a1	2	0.00016	0	0.00000	0.00	53985	9.05	0.2637604	1	2.00%
Q61133	Gstt2	4	0.00032	0	0.00000	0.00	27634	7.02	0.07372955	2	8.60%
P01900	H2-D1	2	0.00016	0	0.00000	0.00	33850	6.38	-0.0905034	4	15.10%
P14429	H2-Q7	7	0.00056	2	0.00021	3.50	37924	5.88	0.0042515	1	3.90%
P14434	H2-Aa	21	0.00167	10	0.00104	2.10	28093	4.64	0.1681641	3	19.90%
P04230	H2-Eb1	3	0.00024	0	0.00000	0.00	30166	5.91	0.02738636	2	9.10%
P08103	Hck	5	0.00040	2	0.00021	2.50	59129	6.82	-0.0417939	3	7.10%
P36552	Cpox	7	0.00056	3	0.00031	2.33	49715	8.81	-0.0752596	3	9.90%
P20060	Hexb	2	0.00016	0	0.00000	0.00	61116	8.28	0.04507461	2	6.70%
O54879	Hmgb3	3	0.00024	0	0.00000	0.00	23010	8.48	-0.3380002	2	16.00%

P14901	Hmox1	3	0.00024	0	0.00000	0.00	32929	6.08	-0.010346	2	12.10%
Q9JIY5	Htra2	2	0.00016	0	0.00000	0.00	49348	9.6	0.06006546	1	2.60%
P49710	Hcls1	19	0.00151	5	0.00052	3.80	54240	4.75	-0.1827157	9	30.00%
P16627	Hspa1l	5	0.00040	2	0.00021	2.50	70637	5.91	-0.0433697	3	6.90%
P13597	Icam1	2	0.00016	0	0.00000	0.00	58844	5.79	0.04495343	2	3.70%
O89110	Casp8	2	0.00016	0	0.00000	0.00	55357	5.12	-0.1167917	2	4.80%
Q9QZD9	Eif3i	7	0.00056	2	0.00021	3.50	36461	5.38	0.00759999	2	7.40%
Q9Z1D1	Eif3g	2	0.00016	0	0.00000	0.00	35638	5.7	-0.1556563	2	14.70%
Q9DCH4	Eif3f	4	0.00032	0	0.00000	0.00	37984	5.33	0.13775636	3	10.50%
Q9DBZ5	Eif3k	2	0.00016	0	0.00000	0.00	25087	4.81	0.05155963	1	6.90%
O55135	Eif6	4	0.00032	0	0.00000	0.00	26511	4.63	0.0922041	2	17.10%
Q9Z1X4	Ilf3	5	0.00040	0	0.00000	0.00	96021	8.86	-0.0761471	3	5.20%
Q9WTQ8	Timm23	4	0.00032	0	0.00000	0.00	21978	9.24	0.15507175	1	4.80%
Q8BKC5	Ipo5	2	0.00016	0	0.00000	0.00	123591	4.82	0.06238837	1	1.00%
Q9EPL8	Ipo7	2	0.00016	0	0.00000	0.00	119486	4.7	0.03477842	2	2.00%
P09055	Itgb1	2	0.00016	0	0.00000	0.00	88231	5.68	-0.0291353	2	4.60%
P11835	Itgb2	17	0.00135	4	0.00042	4.25	85026	7.02	-0.0514656	9	14.80%
Q9D892	Itpa	2	0.00016	0	0.00000	0.00	21897	5.6	0.04585858	1	7.10%
Q9WTP6	Ak2	3	0.00024	0	0.00000	0.00	26469	6.96	-0.0324268	3	15.50%
P05132	Prkaca	4	0.00032	0	0.00000	0.00	40571	8.84	-0.0137892	2	7.70%
P23475	Xrcc6	4	0.00032	0	0.00000	0.00	69484	6.35	-0.1093914	2	3.90%
P62482	Kcnab2	6	0.00048	0	0.00000	0.00	41021	9.11	0.01776566	3	15.00%
Q7TNC4	Luc7l2	2	0.00016	0	0.00000	0.00	46583	10.11	-0.5756882	5	15.30%
O89112	Lancl1	2	0.00016	0	0.00000	0.00	45341	7.88	0.07476193	1	3.00%
P21619	Lmnb2	4	0.00032	0	0.00000	0.00	67318	5.4	-0.2322986	13	30.70%
P32067	Ssb	3	0.00024	0	0.00000	0.00	47756	9.77	-0.2663131	2	7.70%
O08573	Lgals9	7	0.00056	0	0.00000	700.00	40036	9.41	0.05688379	2	7.60%
Q60875	Arhgef2	5	0.00040	2	0.00021	2.50	111974	6.89	-0.152619	3	4.30%
P19973	Lsp1	20	0.00159	8	0.00083	2.50	36714	4.77	-0.2511212	6	28.50%
P97823	Lypla1	3	0.00024	0	0.00000	0.00	24688	6.14	0.16921735	2	17.40%
Q9WTL7	Lypla2	6	0.00048	0	0.00000	0.00	24794	6.74	0.16476196	2	14.30%
P08905	Lyz2	2	0.00016	0	0.00000	0.00	16689	9.11	-0.0616892	3	14.20%
Q9JMH9	Myo18a	30	0.00239	11	0.00114	2.73	232755	5.93	-0.1894485	18	12.40%
P45700	Man1a1	3	0.00024	0	0.00000	0.00	73276	6.34	0.04169461	2	4.30%
Q9QXZ0	Macf1	8	0.00064	3	0.00031	2.67	831878	5.3	-0.1102176	8	1.60%
P31230	Aimp1	2	0.00016	0	0.00000	0.00	33997	8.57	-0.0990968	2	11.00%
Q99MR8	Mccc1	2	0.00016	0	0.00000	0.00	79344	7.69	-0.007099	2	5.40%
P97310	Mcm2	2	0.00016	0	0.00000	0.00	102078	5.49	-0.0985065	2	2.50%
P25206	Mcm3	4	0.00032	0	0.00000	0.00	91546	5.36	-0.1228202	3	7.40%
P49717	Mcm4	2	0.00016	0	0.00000	0.00	96736	6.77	-0.0678074	2	4.20%
Q8VDP3	Mical1	2	0.00016	0	0.00000	0.00	116785	5.73	-0.034208	2	2.30%
P47811	Mapk14	2	0.00016	0	0.00000	0.00	41287	5.55	0.01211114	1	3.10%
P05977	Myl1	8	0.00064	3	0.00031	2.67	20595	4.98	-0.0293617	1	8.50%
Q3THE2	Myl12b	9	0.00072	0	0.00000	900.00	19779	4.71	-0.1352325	6	40.10%
Q8VEM8	Slc25a3	12	0.00095	4	0.00042	3.00	39632	9.36	0.11817933	3	11.20%
Q9CQ65	Mtap	2	0.00016	0	0.00000	0.00	31062	6.71	0.04416962	2	13.40%
P47802	Mtx1	6	0.00048	0	0.00000	0.00	35624	5.82	0.03447953	3	11.40%
Q99104	Myo5a	3	0.00024	0	0.00000	0.00	215538	8.82	-0.1256506	3	1.80%
Q9QY06	Myo9b	3	0.00024	0	0.00000	0.00	238834	8.82	-0.0971005	3	1.50%
O08638	Myh11	33	0.00263	12	0.00125	2.75	227028	5.37	-0.2187219	8	4.10%
Q9ERS2	Ndufa13	5	0.00040	2	0.00021	2.50	16860	9.47	-0.0372917	4	31.90%
P97369	Ncf4	6	0.00048	0	0.00000	0.00	38707	5.95	-0.0416519	5	26.30%
P15532	Nme1	2	0.00016	0	0.00000	0.00	17208	6.84	0.01440789	2	19.10%
Q9CQ75	Ndufa2	2	0.00016	0	0.00000	0.00	10916	10.02	-0.0561616	1	14.10%
P57716	Ncstn	2	0.00016	0	0.00000	0.00	78492	5.75	0.06091812	2	4.00%

Q99LY9	Ndufs5	2	0.00016	0	0.00000	0.00	12648	9.1	-0.2437736	1	11.30%
P32020	Scp2	2	0.00016	0	0.00000	0.00	59126	7.16	0.05544789	2	3.80%
O70378	Cox4nb	2	0.00016	0	0.00000	0.00	12352	9.97	-9.91E-04	1	16.20%
Q61937	Npm1	19	0.00151	8	0.00083	2.38	32560	4.62	-0.1967808	3	21.60%
O55126	Gbas	2	0.00016	0	0.00000	0.00	32933	9.31	-0.069146	2	11.40%
Q9CQE1	Nipsnap3b	4	0.00032	0	0.00000	0.00	28308	9.51	0.01072874	2	8.90%
Q91YT0	Ndufv1	5	0.00040	0	0.00000	0.00	50834	8.51	0.0169181	4	11.90%
Q91WD5	Ndufs2	3	0.00024	0	0.00000	0.00	52626	6.52	0.01114471	2	5.00%
Q99LC3	Ndufa10	5	0.00040	0	0.00000	0.00	40603	7.63	-0.0170141	4	15.80%
Q9DC69	Ndufa9	7	0.00056	3	0.00031	2.33	42525	9.75	0.04912467	2	10.60%
Q9DCT2	Ndufs3	8	0.00064	3	0.00031	2.67	30149	6.67	-0.0426996	6	25.50%
Q8K2K6	Agfg1	2	0.00016	0	0.00000	0.00	58043	8.82	0.05445633	2	9.10%
Q8CHY6	Gatad2a	3	0.00024	0	0.00000	0.00	67334	9.91	-0.0737995	3	7.60%
Q61205	Pafah1b3	2	0.00016	0	0.00000	0.00	25853	6.42	0.02336205	1	3.90%
Q9ERD8	Parvg	3	0.00024	0	0.00000	0.00	37603	5.42	0.03223567	2	11.20%
Q9WU78	Pdcd6ip	5	0.00040	0	0.00000	0.00	96024	6.15	-0.0319563	4	5.40%
Q8BG81	Poldip3	3	0.00024	0	0.00000	0.00	46132	10.06	-0.1094047	2	9.00%
Q9EQ61	Pes1	3	0.00024	0	0.00000	0.00	67796	6.41	-0.1295548	2	4.50%
P23506	Pcmt1	2	0.00016	0	0.00000	0.00	24634	7.1	0.06612332	2	23.30%
Q9CWW6	Pin4	3	0.00024	0	0.00000	0.00	13815	9.78	-0.0570993	2	33.60%
Q60931	Vdac3	6	0.00048	0	0.00000	0.00	30753	8.96	0.04459363	3	15.50%
P53810	Pitpna	3	0.00024	0	0.00000	0.00	31893	5.97	-0.1076384	1	8.50%
P17742	Ppia	19	0.00151	6	0.00062	3.17	17971	7.73	0.0196951	5	35.40%
Q91YR7	Prpf6	5	0.00040	2	0.00021	2.50	106722	8.31	-0.1272472	3	4.10%
P46471	Psmc2	5	0.00040	0	0.00000	0.00	48648	5.72	-0.0761201	4	12.70%
P49722	Psma2	3	0.00024	0	0.00000	0.00	25927	6.91	0.05487181	2	9.40%
Q9R1P3	Psmb2	2	0.00016	0	0.00000	0.00	22906	6.52	0.0160199	2	13.90%
Q9R1P1	Psmb3	2	0.00016	0	0.00000	0.00	22965	6.15	0.08507316	2	14.60%
P26516	Psmd7	9	0.00072	0	0.00000	0.00	36540	6.29	-0.0732087	5	24.00%
Q9D8W5	Psmd12	3	0.00024	0	0.00000	0.00	52895	6.66	-0.089342	2	5.50%
Q64697	Ptprcap	5	0.00040	2	0.00021	2.50	20371	4.43	0.10431473	2	17.80%
Q8R2Y8	Ptrh2	3	0.00024	0	0.00000	0.00	19527	6.95	0.07696129	1	7.70%
Q64455	Ptprj	6	0.00048	0	0.00000	0.00	136769	5.37	0.03694669	4	4.10%
Q64737	Gart	2	0.00016	0	0.00000	0.00	107503	6.25	0.0987825	1	1.10%
P26043	Rdx	10	0.00080	3	0.00031	3.33	68543	5.91	-0.2407373	6	13.40%
P62827	Ran	9	0.00072	4	0.00042	2.25	24423	7.01	0.02361109	2	6.90%
Q8K386	Rab15	2	0.00016	0	0.00000	0.00	24318	5.5	-0.0982547	1	5.20%
P35293	Rab18	3	0.00024	0	0.00000	0.00	23035	5.24	-0.0341748	2	12.60%
P35282	Rab21	7	0.00056	3	0.00031	2.33	24106	8.11	-0.0279279	4	22.10%
P35279	Rab6a	3	0.00024	0	0.00000	0.00	23590	5.42	-0.0762019	3	21.60%
Q9R0M6	Rab9a	2	0.00016	0	0.00000	0.00	22910	5.44	-0.0603483	1	5.50%
P15307	Rel	2	0.00016	0	0.00000	0.00	64960	6.1	-0.044276	1	4.90%
Q9WUK4	Rfc2	7	0.00056	0	0.00000	0.00	38725	6.04	0.03406876	4	14.60%
P46061	Rangap1	4	0.00032	0	0.00000	0.00	63531	4.59	0.02483871	3	9.20%
Q9CPR4	Rpl17	9	0.00072	4	0.00042	2.25	21423	10.2	-0.2566848	6	26.10%
P67984	Rpl22	9	0.00072	3	0.00031	3.00	14759	9.21	-0.1804687	2	21.90%
P47962	Rpl5	7	0.00056	0	0.00000	0.00	34401	9.78	-0.1918182	3	12.80%
P12970	Rpl7a	7	0.00056	2	0.00021	3.50	29977	10.56	-0.1664285	2	10.20%
P14148	Rpl7	10	0.00080	3	0.00031	3.33	31420	10.89	-0.1862221	5	20.70%
P49312	Hnrnpa1	9	0.00072	3	0.00031	3.00	34196	9.27	-0.1165314	4	13.40%
O88569	Hnrnpa2b1	29	0.00231	12	0.00125	2.42	37403	8.97	-0.0845327	8	35.70%
Q8BG05	Hnrnpa3	14	0.00111	5	0.00052	2.80	39652	9.1	-0.1065701	4	12.90%
P70336	Rock2	10	0.00080	2	0.00021	5.00	160586	5.73	-0.1973563	5	5.70%
Q9CRA8	Exosc5	2	0.00016	0	0.00000	0.00	25194	7.52	0.02668085	2	9.80%
P62071	Rab21	7	0.00056	3	0.00031	2.33	24106	8.11	-0.0279279	4	22.10%

P63323	Rps12	3	0.00024	0	0.00000	0.00	14525	6.82	-0.0247727	1	7.60%
Q9CZX8	Rps19	9	0.00072	3	0.00031	3.00	16085	10.41	-0.1603448	5	35.20%
Q9CQR2	Rps21	3	0.00024	0	0.00000	0.00	9141	8.71	-0.0810843	2	28.90%
P25444	Rps2	6	0.00048	0	0.00000	0.00	31231	10.25	0.00477815	3	11.60%
P97351	Rps3a	5	0.00040	0	0.00000	0.00	29885	9.75	-0.1451516	3	17.00%
P14206	Rpsa	22	0.00175	9	0.00093	2.44	32838	4.8	0.04132203	7	29.50%
Q9ER88	Dap3	2	0.00016	0	0.00000	0.00	44699	9.1	-0.0461637	1	3.10%
Q62189	Snrpa	15	0.00119	7	0.00073	2.14	31835	9.81	0.01533101	5	25.40%
Q62241	Snrpc	2	0.00016	0	0.00000	0.00	17364	9.72	-0.00239	2	13.20%
P70122	Sbds	3	0.00024	0	0.00000	0.00	28781	8.92	-0.1389601	1	8.80%
Q9ERN0	Scamp2	2	0.00016	0	0.00000	0.00	36465	5.94	0.17699091	2	9.70%
Q8C1B7	Sep-11	10	0.00080	4	0.00042	2.50	49695	6.24	-0.1391415	5	13.50%
Q9BCZ4	Vimp	2	0.00016	0	0.00000	0.00	21359	9.44	-0.2109524	1	11.10%
P42209	Sep-1	11	0.00088	4	0.00042	2.75	42020	5.59	-0.1382787	7	22.40%
O55131	Sep-7	10	0.00080	3	0.00031	3.33	50550	8.73	-0.1795413	4	15.80%
Q8BRF7	Scfd1	3	0.00024	0	0.00000	0.00	72323	5.98	-0.0172614	3	7.00%
Q8VE97	Sfrs4	5	0.00040	2	0.00021	2.50	55979	11.39	-0.649508	1	2.70%
O35326	Sfrs5	3	0.00024	0	0.00000	0.00	30891	11.56	-0.5140517	2	8.90%
Q8CG47	Smc4	3	0.00024	0	0.00000	0.00	146895	6.91	-0.1478307	2	2.00%
O09044	Snap23	13	0.00103	5	0.00052	2.60	23261	4.88	-0.187	4	35.70%
P09671	Sod2	7	0.00056	2	0.00021	3.50	24603	8.8	0.02554055	4	26.60%
Q9QX47	Son	8	0.00064	3	0.00031	2.67	265651	5.46	-0.0865426	5	3.20%
O55128	Sap18	2	0.00016	0	0.00000	0.00	17595	9.38	-0.1649673	1	10.50%
O89090	Sp1	2	0.00016	0	0.00000	0.00	80732	6.94	0.01920915	1	3.60%
P46062	Sipa1	10	0.00080	4	0.00042	2.50	112066	5.94	-0.0376664	7	10.90%
P49962	Srp9	2	0.00016	0	0.00000	0.00	10194	7.78	-0.1204651	1	12.80%
Q60770	Stxbp3	4	0.00032	0	0.00000	0.00	67942	8.28	-0.0843074	3	5.20%
O55098	Stk10	15	0.00119	6	0.00062	2.50	111906	6.71	-0.2537063	5	6.30%
P54116	Stom	2	0.00016	0	0.00000	0.00	31375	6.46	0.0657042	2	10.90%
Q9EPQ7	Stard5	2	0.00016	0	0.00000	0.00	23922	5.97	-0.0206573	1	5.20%
Q93092	Taldo1	4	0.00032	0	0.00000	0.00	37387	6.57	-0.0067656	3	10.10%
P36371	Tap2	6	0.00048	0	0.00000	0.00	77445	6.82	0.12984376	5	9.80%
Q61390	Cct6b	5	0.00040	2	0.00021	2.50	58185	6.96	0.04625235	1	4.00%
Q62393	Tpd52	5	0.00040	0	0.00000	0.00	24313	4.69	-0.0802232	3	24.60%
Q9CQR4	Acot13	3	0.00024	0	0.00000	0.00	15183	8.95	0.0792857	2	15.00%
P62915	Gtf2b	2	0.00016	0	0.00000	0.00	34819	8.67	-0.0548418	1	4.40%
P40630	Tfam	21	0.00167	10	0.00104	2.10	27988	9.71	-0.1893005	8	33.30%
P70318	Tial1	2	0.00016	0	0.00000	0.00	43389	8.11	0.01112243	1	5.90%
Q8C0G2	Traf3ip3	2	0.00016	0	0.00000	0.00	58564	8.46	-0.2357895	2	6.20%
P21107	Tpm3	2	0.00016	0	0.00000	0.00	32994	4.68	-0.291579	1	3.90%
P35441	Thbs1	7	0.00056	0	0.00000	700.00	129647	4.72	-0.1092392	4	4.00%
Q9WUP7	Uchl5	5	0.00040	2	0.00021	2.50	37617	5.24	-0.0364134	5	22.20%
Q9CR68	Uqcrfs1	6	0.00048	0	0.00000	0.00	29368	8.92	0.03974452	4	24.50%
P50516	Atp6v1a	10	0.00080	3	0.00031	3.33	68326	5.42	0.03726094	9	22.90%
Q9WV55	Vapa	9	0.00072	4	0.00042	2.25	27855	8.58	-0.0543775	3	18.90%
Q9QY76	Vapb	2	0.00016	0	0.00000	0.00	26946	7.64	-0.0505762	2	10.70%
P50518	Atp6v1e1	2	0.00016	0	0.00000	0.00	26157	8.44	-0.1374337	2	11.90%
Q9DBH5	Lman2	5	0.00040	0	0.00000	0.00	40430	6.46	0.01159217	3	12.60%
Q91VU6	Dcaf11	2	0.00016	0	0.00000	0.00	61992	6	-0.1051002	1	3.30%
Q6ZQL4	Wdr43	2	0.00016	0	0.00000	0.00	75381	5.23	-0.0694092	1	4.10%
O88342	Wdr1	11	0.00088	2	0.00021	5.50	66407	6.11	0.05250826	6	18.00%
P83741	Wnk1	2	0.00016	0	0.00000	0.00	250934	6	-0.000829	1	0.50%
Q9CYH2	Fam213a	2	0.00016	0	0.00000	0.00	24395	9.22	0.03899083	2	11.90%
Q99JY4	Trabd	2	0.00016	0	0.00000	0.00	42189	8.65	-0.0244947	1	3.20%
Q9QY24	Zbp1	2	0.00016	0	0.00000	0.00	44331	5.47	-0.0361314	1	3.20%

O08692	Ngp	2	0.00016	0	0.00000	0.00	19332	5.21	-0.1941917	1	12.60%
Q9QUG9	Rasgrp2	2	0.00016	0	0.00000	0.00	69446	7.6	-0.044227	1	3.10%
O35405	Pld3	2	0.00016	0	0.00000	0.00	54389	6.07	0.07581967	2	4.50%
A1L333	Ddx5	5	0.00040	2	0.00021	2.50	69290	9.06	-0.1188434	10	27.40%
Q794H2	Nap1l3	2	0.00016	0	0.00000	0.00	61377	4.74	-0.192463	1	2.60%
O70145	Ncf2	4	0.00032	0	0.00000	0.00	59485	6.18	-0.0282857	2	7.40%
Q8CDN6	Txnl1	2	0.00016	0	0.00000	0.00	32237	4.84	-0.0224568	1	5.20%
Q52KI8	Srrm1	3	0.00024	0	0.00000	0.00	106862	11.87	-0.5358872	3	4.40%
P62264	Rps14	9	0.00072	2	0.00021	4.50	16273	10.07	-0.1370199	2	10.60%
Q9QWR8	Naga	5	0.00040	2	0.00021	2.50	47235	6.02	0.07489154	3	9.90%
P70388	Rad50	2	0.00016	0	0.00000	0.00	153488	6.53	-0.2609597	2	1.80%
P70670	Naca	4	0.00032	0	0.00000	0.00	220499	9.39	0.03425754	2	1.40%
Q31150	H2-Oa	4	0.00032	0	0.00000	0.00	28167	6.44	0.11312003	2	12.80%
Q31188	MHC integra	2	0.00016	0	0.00000	0.00	46976	8.65	-0.0017577	2	15.20%
Q60749	Khdrbs1	3	0.00024	0	0.00000	0.00	48371	8.81	-0.1111513	1	3.20%
Q60766	Irgm	8	0.00064	2	0.00021	4.00	72585	7.06	0.01418673	6	19.70%
Q61107	Gbp4	5	0.00040	0	0.00000	0.00	70801	6.23	-0.0638387	2	4.20%
O35129	Phb2	14	0.00111	6	0.00062	2.33	33296	9.83	-0.0453846	8	29.80%
Q3U0K0	Ifi47	5	0.00040	2	0.00021	2.50	46785	6.28	0.08076191	4	13.10%
Q61735	Cd47	9	0.00072	4	0.00042	2.25	33098	8.93	0.27831697	2	8.90%
Q3TTY5	Krt2	3	0.00024	0	0.00000	0.00	70923	8.26	-0.0205376	3	3.70%
Q61769	Mki67	3	0.00024	0	0.00000	0.00	350864	9.73	-0.2536632	2	2.00%
Q64735	Crry	2	0.00016	0	0.00000	0.00	36544	4.89	-0.0244947	1	3.20%
P14131	Rps16	5	0.00040	2	0.00021	2.50	16445	10.21	-0.1269178	4	29.50%
P63242	Eif5a	3	0.00024	0	0.00000	0.00	16832	5.07	0.01058442	2	8.40%
Q6IFZ6	Krt77	4	0.00032	0	0.00000	0.00	61359	7.74	-0.0684965	1	2.10%
Q68FL4	Ahcyl2	3	0.00024	0	0.00000	0.00	66899	7.13	-0.0092659	2	5.90%
Q3U2N8	Wdfy4	2	0.00016	0	0.00000	0.00	337363	5.94	0.07107451	2	0.80%
Q8BLF1	Nceh1	2	0.00016	0	0.00000	0.00	45740	6.56	0.09843139	1	2.90%
Q925I1	Atad3	10	0.00080	3	0.00031	3.33	66742	9.32	-0.1640267	7	14.20%
Q69ZN7	Myof	5	0.00040	0	0.00000	0.00	233324	5.83	-0.0386718	5	3.30%
Q8C7E9	Cstf2t	2	0.00016	0	0.00000	0.00	65862	6.78	0.00522151	2	8.20%
Q3TBU0	Unc84b	2	0.00016	0	0.00000	0.00	#N/A	#N/A	#N/A	#N/A	#N/A
Q3UVT5	Pi4ka	8	0.00064	0	0.00000	0.00	231355	6.44	0.04967243	6	3.70%
Q8BH95	Echs1	3	0.00024	0	0.00000	0.00	31474	8.76	0.03020692	2	8.60%
Q3UFZ6	Caprin1	2	0.00016	0	0.00000	0.00	78169	5.14	-0.1408908	4	7.50%
Q4VBF6	Bcl2	2	0.00016	0	0.00000	0.00	26407	6.65	0.07419491	1	11.40%
Q6NV83	U2surp	2	0.00016	0	0.00000	0.00	118261	8.6	-0.2900579	1	1.20%
Q6NZB0	Dnajc8	6	0.00048	2	0.00021	3.00	29813	9.04	-0.3714228	5	20.20%
Q6NZJ6	Eif4g1	2	0.00016	0	0.00000	0.00	176077	5.3	-0.0947565	5	5.60%
Q6P4S8	Ints1	2	0.00016	0	0.00000	0.00	245168	5.87	0.0109385	2	1.30%
Q6P5E6	Gga2	2	0.00016	0	0.00000	0.00	66049	6.88	0.03771146	1	3.00%
Q5H8C4	Vps13a	2	0.00016	0	0.00000	0.00	359401	5.83	0.03904932	1	0.50%
Q640N3	Arhgap30	2	0.00016	0	0.00000	0.00	120114	4.79	-0.1090643	5	5.40%
Q6P9J9	Ano6	2	0.00016	0	0.00000	0.00	106255	6.34	0.07382003	2	3.50%
Q3UFI7	Nup205	3	0.00024	0	0.00000	0.00	227465	5.92	0.03162362	3	2.80%
Q6P9R2	Oxsr1	3	0.00024	0	0.00000	0.00	58214	6.03	-0.0260531	2	4.70%
A2AN08	Ubr4	3	0.00024	0	0.00000	0.00	572290	5.72	0.02410425	2	0.50%
Q6PE01	Snrnp40	11	0.00088	3	0.00031	3.67	39276	8.34	0.00234636	5	30.70%
O08585	Clta	2	0.00016	0	0.00000	0.00	25604	4.5	-0.0944255	1	8.90%
Q6ZQ38	Cand1	4	0.00032	0	0.00000	0.00	136332	5.52	0.04928459	3	3.10%
Q3UHQ5	Gcn1l1	3	0.00024	0	0.00000	0.00	293021	7.14	0.06586672	5	2.20%
Q9QXS1	Plec	40	0.00318	16	0.00166	2.50	534216	5.74	-0.183927	35	9.70%
Q78HU7	Gypc	3	0.00024	0	0.00000	0.00	10329	5.21	0.15463157	1	28.40%
Q6ZPE2	Sbf1	2	0.00016	0	0.00000	0.00	208693	6.71	-0.0247723	1	0.90%

Q6ZQ08	Cnot1	3	0.00024	0	0.00000	0.00	266808	6.65	0.05032018	5	2.50%
Q8BG67	Efr3a	2	0.00016	0	0.00000	0.00	92613	6.36	0.00568988	1	1.70%
P61327	Magoh	2	0.00016	0	0.00000	0.00	17164	5.74	-0.044726	2	26.00%
Q9CPV4	Glod4	2	0.00016	0	0.00000	0.00	33317	5.28	-0.0160738	1	6.00%
Q7TN13	Cad	2	0.00016	0	0.00000	0.00	243238	6	0.07306494	9	6.10%
Q7TN29	Smap2	2	0.00016	0	0.00000	0.00	46578	9.01	0.00341121	2	9.60%
Q7TNS7	Asic4	2	0.00016	0	0.00000	0.00	59216	6.32	0.02146566	1	3.20%
Q7TPH6	Mycbp2	2	0.00016	0	0.00000	0.00	517738	6.75	-0.0184949	1	0.30%
P83917	Cbx1	2	0.00016	0	0.00000	0.00	21418	4.85	-0.2960541	1	9.20%
Q7TSA3	Btla	5	0.00040	2	0.00021	2.50	34337	7.11	-0.0160784	1	3.90%
Q5DTM8	Rnf20	2	0.00016	0	0.00000	0.00	113520	5.74	-0.31001	1	1.10%
Q3TLH4	Prrc2c	3	0.00024	0	0.00000	0.00	310892	9.14	-0.196289	1	0.60%
P26039	Tln1	3	0.00024	0	0.00000	0.00	269821	5.84	-0.0027587	55	34.00%
Q80TU6	Acin1	2	0.00016	0	0.00000	0.00	150719	5.71	-0.3573538	7	8.70%
Q8BTI8	Srrm2	5	0.00040	0	0.00000	0.00	294840	12.02	-0.4453489	11	6.70%
Q80V42	Cpm	7	0.00056	0	0.00000	0.00	50556	7.66	0.04503384	4	12.00%
A2AR02	Ppig	2	0.00016	0	0.00000	0.00	88325	10.27	-0.6307033	1	2.40%
Q80VD1	Fam98b	3	0.00024	0	0.00000	0.00	45349	8.77	-0.0531236	2	6.50%
Q80WS3	Fbll1	2	0.00016	0	0.00000	0.00	33339	10.16	-0.0502548	1	3.50%
Q8C2K1	Def6	9	0.00072	4	0.00042	2.25	73454	5.94	-0.2358888	4	7.80%
P62702	Rps4x	5	0.00040	2	0.00021	2.50	29598	10.16	-0.0631179	3	7.20%
Q5SUA5	Myo1g	21	0.00167	10	0.00104	2.10	117227	8.83	-0.0725976	23	28.60%
Q3TIX9	Usp39	2	0.00016	0	0.00000	0.00	65146	9.01	-0.1102836	1	2.10%
Q8BFZ9	Erlin2	2	0.00016	0	0.00000	0.00	37873	5.37	0.05073532	2	7.10%
Q9EQ32	Pik3ap1	2	0.00016	0	0.00000	0.00	90928	5.06	-0.0724784	1	1.50%
Q8BPF4	Hba-a1	5	0.00040	0	0.00000	0.00	15112	7.97	0.11295774	2	21.10%
Q8CCF0	Prpf31	5	0.00040	0	0.00000	0.00	55430	5.55	-0.0778156	3	12.60%
Q8BP67	Rpl24	2	0.00016	0	0.00000	0.00	17779	11.26	-0.3231211	1	7.60%
P62754	Rps6	3	0.00024	0	0.00000	0.00	28681	10.85	-0.3571887	2	10.40%
P61979	Hnrnpk	9	0.00072	4	0.00042	2.25	50976	5.39	-0.0940389	5	9.70%
P63276	Rps17	6	0.00048	2	0.00021	3.00	15524	9.85	-0.1668889	1	16.30%
Q66K09	5830443L24	2	0.00016	0	0.00000	0.00	14099	11.47	-0.4452032	1	11.40%
Q8BTX9	Hsdl1	4	0.00032	0	0.00000	0.00	36868	8.73	0.12842429	3	18.20%
Q8BV28	Coq10a	2	0.00016	0	0.00000	0.00	65146	9.01	-0.1102836	1	2.10%
Q9CQT1	Mri1	2	0.00016	0	0.00000	0.00	39411	5.6	0.0785908	1	5.70%
Q4V9U6	Gimap9	4	0.00032	0	0.00000	0.00	15494	8.61	-0.0010145	1	10.10%
Q5F2E8	Taok1	2	0.00016	0	0.00000	0.00	116050	7.14	-0.2106896	2	2.10%
Q8BPB0	Mob1b	2	0.00016	0	0.00000	0.00	25091	6.24	0.0275926	1	5.60%
P62141	Ppp1cb	3	0.00024	0	0.00000	0.00	37187	5.84	0.00198776	1	3.10%
Q9CZX0	Elp3	2	0.00016	0	0.00000	0.00	62385	9.05	-0.0453017	1	2.90%
Q8C2Q3	Rbm14	3	0.00024	0	0.00000	0.00	69449	9.68	-0.0026607	7	17.80%
Q922Q1	MOSC2	2	0.00016	0	0.00000	0.00	38194	8.95	-0.0318639	2	7.70%
Q505D7	Opa3	2	0.00016	0	0.00000	0.00	20110	8.62	-0.0190503	1	6.70%
Q8C7R4	Uba6	2	0.00016	0	0.00000	0.00	117966	5.75	0.02835709	1	1.30%
Q8VDL4	Adpgk	2	0.00016	0	0.00000	0.00	53902	5.37	0.13447587	2	5.80%
A2AF47	Dock11	4	0.00032	0	0.00000	0.00	237771	7.94	-0.0229715	2	0.90%
Q9CXY6	Ilf2	3	0.00024	0	0.00000	0.00	43062	5.19	0.02479488	3	12.60%
P70227	Itpr3	2	0.00016	0	0.00000	0.00	304275	6.11	-0.0050711	2	1.00%
Q3U6Q5	Lyn	2	0.00016	0	0.00000	0.00	58812	6.74	-0.0661719	13	32.60%
P63280	Ube2i	3	0.00024	0	0.00000	0.00	18007	8.87	-0.0411393	2	16.50%
Q8CI43	Myl6b	12	0.00095	3	0.00031	4.00	22749	5.41	-0.0634783	1	4.30%
Q8BGU5	Ccny	3	0.00024	0	0.00000	0.00	39395	6.76	-0.1043695	2	9.40%
Q9D2G2	Dlst	6	0.00048	2	0.00021	3.00	48995	9.1	0.00944932	1	2.60%
Q8JZQ2	Afg3l2	2	0.00016	0	0.00000	0.00	89519	8.77	-0.0341771	1	1.70%
Q8K051	Trip12	3	0.00024	0	0.00000	0.00	224128	8.64	-0.0783655	2	1.30%

Q8K124	Plekho2	3	0.00024	0	0.00000	0.00	53872	5.4	-0.1549697	2	9.10%
Q8K1M6	Dnm1l	4	0.00032	0	0.00000	0.00	82658	6.61	-0.0342049	4	7.70%
Q8K4G5	Ablim1	2	0.00016	0	0.00000	0.00	96805	8.93	-0.1296166	2	4.40%
Q8K2H4	Acap1	9	0.00072	0	0.00000	900.00	81704	7.32	-0.0522703	5	13.80%
Q8K2Q7	Brox	2	0.00016	0	0.00000	0.00	46202	7.59	-0.0133333	1	2.70%
Q8R010	Aimp2	2	0.00016	0	0.00000	0.00	35378	7.7	0.04068749	1	7.80%
Q8R0B3	Armcx5	2	0.00016	0	0.00000	0.00	46489	6.47	0.06974179	1	4.70%
Q8R0F6	Ilkap	2	0.00016	0	0.00000	0.00	42774	7.04	-0.0830612	2	5.40%
Q91ZJ5	Ugp2	2	0.00016	0	0.00000	0.00	56979	7.18	0.00635827	2	4.50%
Q8CHT0	Aldh4a1	2	0.00016	0	0.00000	0.00	61841	8.45	0.04590746	2	4.60%
Q569Z5	Ddx46	5	0.00040	0	0.00000	0.00	117448	9.3	-0.2657756	5	5.90%
Q5ND34	Wdr81	2	0.00016	0	0.00000	0.00	211945	5.5	0.02994831	1	0.60%
Q5XJY5	Arcn1	2	0.00016	0	0.00000	0.00	57229	5.89	-0.0435813	2	8.20%
Q9DBA8	Amdhd1	2	0.00016	0	0.00000	0.00	46489	6.47	0.06974179	1	4.70%
Q8R2K3	Ssbp1	2	0.00016	0	0.00000	0.00	17157	9.78	-0.1196622	1	12.20%
Q80UN3	Nup153	2	0.00016	0	0.00000	0.00	152008	9.04	-0.0073256	1	2.00%
Q8BL97	Sfrs7	5	0.00040	0	0.00000	0.00	30818	11.89	-0.4581271	2	9.70%
Q3TKT4	Smarca4	7	0.00056	3	0.00031	2.33	181427	8	-0.1596217	8	8.20%
Q8VDW0	Ddx39a	6	0.00048	0	0.00000	0.00	49067	5.46	-0.0474473	9	22.20%
Q8VE10	Naa40	2	0.00016	0	0.00000	0.00	27229	7.03	-0.1224895	1	5.10%
A2AW41	Hnrnpr	10	0.00080	3	0.00031	3.33	30064	5.24	-0.0460741	4	26.30%
Q80VQ0	Aldh3b1	2	0.00016	0	0.00000	0.00	52292	7.5	0.02269231	1	3.00%
Q1AFZ1	EG667622	10	0.00080	3	0.00031	3.33	66742	9.32	-0.1640267	7	14.20%
Q91V64	Isoc1	3	0.00024	0	0.00000	0.00	32033	6.96	0.17993271	3	13.80%
Q91W98	Slc15a4	2	0.00016	0	0.00000	0.00	62256	9.32	0.23015682	1	2.30%
Q91WN4	Kmo	2	0.00016	0	0.00000	0.00	54532	9.07	0.06830896	2	4.20%
Q8BMF4	Dlat	7	0.00056	3	0.00031	2.33	67942	8.81	0.08733644	4	11.20%
Q4VAA2	Cdv3	2	0.00016	0	0.00000	0.00	29729	5.84	-0.2417794	1	8.20%
Q5SUF2	Luc7l3	3	0.00024	0	0.00000	0.00	51450	9.79	-0.6118047	2	7.40%
Q91VR5	Ddx1	3	0.00024	0	0.00000	0.00	82500	6.8	-0.015446	2	4.10%
Q922Q8	Lrrc59	6	0.00048	2	0.00021	3.00	34877	9.57	-0.2306188	3	12.10%
Q91VI7	Rnh1	2	0.00016	0	0.00000	0.00	49816	4.69	0.02986843	1	2.60%
Q3T998	Akap13	2	0.00016	0	0.00000	0.00	303976	5.26	-0.124618	1	0.40%
Q31096	H2-DMa	2	0.00016	0	0.00000	0.00	29056	4.6	0.16854411	1	8.00%
Q9CY50	Ssr1	2	0.00016	0	0.00000	0.00	32065	4.36	-0.000909	2	14.30%
Q99N16	Cyp4f3	3	0.00024	0	0.00000	0.00	59843	8.82	0.04135498	1	2.10%
Q9CZM2	Rpl15	2	0.00016	0	0.00000	0.00	24146	11.62	-0.2967157	2	13.20%
A8DUK2	Hbb-b1	11	0.00088	2	0.00021	5.50	15840	7.13	0.14292522	5	47.60%
Q9JHS9	Cwc15	3	0.00024	0	0.00000	0.00	26624	5.54	-0.421048	2	11.40%
Q9D1J1	Necap2	3	0.00024	0	0.00000	0.00	28598	7.72	-0.0177068	2	13.90%
Q9CX86	Hnrnpa0	5	0.00040	0	0.00000	0.00	30530	9.35	0.03734424	3	14.10%
Q9CXD6	Mcur1	3	0.00024	0	0.00000	0.00	37849	10.21	-0.0686765	2	7.40%
Q9JIY0	Plekho1	3	0.00024	0	0.00000	0.00	45997	9.08	-0.2460048	2	8.60%
P63321	Rala	5	0.00040	0	0.00000	0.00	23553	6.66	-0.1676214	4	20.90%
P62900	Rpl31	6	0.00048	0	0.00000	0.00	14463	10.54	-0.2620801	2	18.40%
Q78IK4	Apool	6	0.00048	0	0.00000	0.00	29261	9.34	0.00566036	3	16.20%
Q8R344	Ccdc12	2	0.00016	0	0.00000	0.00	18891	6.69	-0.380241	1	15.10%
Q9ESW4	Agk	2	0.00016	0	0.00000	0.00	46976	8.65	-0.0017577	3	15.20%
Q9D3W2	Speer4d	2	0.00016	0	0.00000	0.00	25002	5.24	-0.196085	1	6.10%
Q9D967	Mdp1	2	0.00016	0	0.00000	0.00	18582	6.29	0.02774389	1	7.90%
Q9CQW2	Arl8b	4	0.00032	0	0.00000	0.00	21539	8.67	-0.0345161	1	4.80%
Q8VE70	Pdcd10	9	0.00072	4	0.00042	2.25	24716	7.8	-0.0826887	6	33.50%
P42125	Dci1	3	0.00024	0	0.00000	0.00	32250	9.12	0.00844293	4	20.40%
Q3U2S8	Hvcn1	10	0.00080	4	0.00042	2.50	31242	6.57	0.06159845	5	24.20%
Q9DCE9	Igtp	2	0.00016	0	0.00000	0.00	48494	7.63	-0.0291253	3	10.20%

Q9DCS3	Mecr	2	0.00016	0	0.00000	0.00	40343	9.17	0.06664876	1	4.00%
P68040	Gnb2l1	2	0.00016	0	0.00000	0.00	35077	7.6	0.03943218	1	7.60%
Q9ERU9	Ranbp2	2	0.00016	0	0.00000	0.00	341121	5.82	-0.082378	5	2.00%
Q9JM90	Stap1	2	0.00016	0	0.00000	0.00	34628	8.55	-0.1051515	1	7.70%
P62918	Rpl8	5	0.00040	2	0.00021	2.50	28025	11.04	-0.1378211	3	14.40%

S4 Table: The list of reduced proteins in gp96 KO B cells.

443 of proteins down-regulated in gp96 KO B cells were selected from 1425 probes based on MS/MS results. The selection cutoff was WT/KO larger or equal than 2. Enrichment GO annotation was done after
