# Supplementary material for: Mapping the Interactome of a Major Mammalian Endoplasmic Reticulum Heat Shock Protein 90
Source: PLoS One. 2017 Jan 5;12(1):e0169260. doi: 10.1371/journal.pone.0169260 (PMC5215799; doi:10.1371/journal.pone.0169260)
Supplement: S5 Table — 23 of proteins up-regulated in gp96 KO B cells were selected from 1425 probes based on MS/MS results. The selection cutoff was WT/KO less or equal than 0.5. (PDF) [file pone.0169260.s005.pdf]

S5 Table: Increased proteins in gp96 KO B cells

Accession	Gene Symbol	WT peptide	Normalized SC.wt.	KO Peptide	Normalized SC.ko.	WT/KO ratio	MW	pI	Hydro	Unique Peptide	aa Coverage
P10107	Anxa1	3	0.00024	7	0.00073	0.43	38734	6.97	-0.07419074	5	16.20%
Q99MR6	Srrt	4	0.00032	9	0.00093	0.44	100452	5.71	-0.2568577	5	8.00%
P08553	Nefm	4	0.00032	9	0.00093	0.44	95916	4.76	-0.27930462	1	1.40%
Q99KV1	Dnajb11	3	0.00024	7	0.00073	0.43	40555	5.92	-0.08215086	2	8.40%
Q9Z0N1	Eif2s3x	2	0.00016	6	0.00062	0.33	51065	8.66	0.06851699	4	13.80%
P48678	Lmna	4	0.00032	19	0.00197	0.21	74238	6.54	-0.24807513	12	25.60%
E9Q634	Myo1e	2	0.00016	5	0.00052	0.40	126818	9.14	-0.07691952	8	8.40%
P15331	Prph	2	0.00016	7	0.00073	0.29	54268	5.4	-0.22035775	2	4.40%
Q04750	Top1	2	0.00016	6	0.00062	0.33	90876	9.35	-0.34001336	4	6.80%
Q80X41	Vrk1	2	0.00016	6	0.00062	0.33	49741	9.08	-0.14990911	5	15.50%
Q61191	Hcfc1	2	0.00016	5	0.00052	0.40	210437	6.81	0.10296742	4	2.70%
Q6IFX2	Krt42	5	0.00040	12	0.00125	0.42	50133	5.09	-0.11466812	3	6.90%
Q3UMY5	Eml4	5	0.00040	11	0.00114	0.45	110027	6.08	-0.05358297	6	8.00%
Q80VH0	Bank1	2	0.00016	5	0.00052	0.40	89406	5.47	-0.13191563	4	8.60%
Q9JKR6	Hyou1	3	0.00024	7	0.00073	0.43	111181	5.12	-0.09438428	7	11.80%
Q63ZW9	Copa	3	0.00024	9	0.00093	0.33	138432	7.69	-0.00108659	9	10.40%
Q9Z315	Sart1	2	0.00016	6	0.00062	0.33	90885	5.66	-0.29457852	5	9.60%
Q6A068	Cdc5l	2	0.00016	5	0.00052	0.40	92190	7.98	-0.24003759	3	4.70%
Q6PB66	Lrpprc	6	0.00048	14	0.00145	0.43	156615	6.42	0.006135076	10	9.10%
P62320	Snrpd3	2	0.00016	6	0.00062	0.33	13916	10.33	-0.1081746	2	15.10%
Q922U2	Krt5	4	0.00032	9	0.00093	0.44	61767	7.59	-0.03617247	4	10.20%
Q99JF8	Psip1	2	0.00016	5	0.00052	0.40	59697	9.15	-0.3593182	6	12.10%
A2AFQ2	Hsd17b10	3	0.00024	8	0.00083	0.38	27419	8.53	0.13521077	2	19.50%

S5 Table: Increased proteins in gp96 KO B cells. 23 of proteins up-regulated in gp96 KO B cells were selected from 1425 probes based on MS/MS results. The selection cutoff was WT/KO less or equal than 0.5.
