# Supplementary material for: Mapping the Interactome of a Major Mammalian Endoplasmic Reticulum Heat Shock Protein 90
Source: PLoS One. 2017 Jan 5;12(1):e0169260. doi: 10.1371/journal.pone.0169260 (PMC5215799; doi:10.1371/journal.pone.0169260)
Supplement: S6 Table — 811 clean entries were detected from Immunoprecipitation method. A total of 201 genes were selected from those 811 genes with expectation value small than 0.05. (PDF) [file pone.0169260.s006.pdf]

S6 Table: gp96-associated proteins in preB cells

name	proteinID	Protein description	score	expectation	MW	coverage	empai
Birc6	IPI00134095	Birc6 baculoviral IAP repeat-containing 6	211	4.80E-17	529085	2.3	0.04
Lamc1	IPI00400016	Lamc1 Laminin subunit gamma-1	399	7.60E-36	177185	10.7	0.33
Uggt1	IPI00762897	Uggt1 UDP-glucose:glycoprotein glucosyltransferase 1	1965	0	176322	43.4	3.25
Pzp	IPI00624663	Pzp Putative uncharacterized protein Pzp	340	5.30E-30	167177	14.5	0.39
Mug1	IPI00123223	Mug1 Murinoglobulin-1	86	0.00014	165193	0.9	0.03
Dsp	IPI00553419	Dsp desmoplakin	540	5.40E-50	332706	14.4	0.36
Man2b1	IPI00381303	Man2b1 Lysosomal alpha-mannosidase	380	5.20E-34	114532	15.4	0.38
Ganab	IPI00115679	Ganab Isoform 2 of Neutral alpha-glucosidase AB	310	6.00E-27	109335	19.8	0.53
Hyou1	IPI00123342	Hyou1 Hypoxia up-regulated protein 1	264	2.30E-22	111112	27.2	0.4
Lrpprc	IPI00420706	Lrpprc Leucine-rich PPR motif-containing protein, mitochondrial	144	2.30E-10	156516	5.4	0.16
Hsp90ab1	IPI00229080	Hsp90ab1 Putative uncharacterized protein	595	1.80E-55	83229	31.9	1.57
Plod3	IPI00127406	Plod3 Procollagen-lysine,2-oxoglutarate 5-dioxygenase 3	480	6.20E-44	84869	27.5	1.26
Hsp90aa1	IPI00330804	Hsp90aa1 Heat shock protein HSP 90-alpha	366	1.50E-32	84735	16.8	0.46
Trf	IPI00139788	Trf Serotransferrin	352	3.60E-31	76674	18.2	0.94
Plod1	IPI00127407	Plod1 Procollagen-lysine,2-oxoglutarate 5-dioxygenase 1	314	2.10E-27	83542	20.3	0.74
Hspa5	IPI00319992	Hspa5 78 kDa glucose-regulated protein	4919	0	72377	68.9	224.28
Pdia4	IPI00271951	Pdia4 protein disulfide-isomerase A4	2189	0	72325	61.2	32.29
Hspa8	IPI00323357	Hspa8 Heat shock cognate 71 kDa protein	1057	1.10E-101	70827	36.4	3.19
Glt25d1	IPI00169870	Glt25d1 Procollagen galactosyltransferase 1	347	1.10E-30	71015	31.4	1.33
Hspa9	IPI00133903	Hspa9 Stress-70 protein, mitochondrial	274	2.10E-23	73483	19.3	0.87
Clpx	IPI00119808	Clpx ATP-dependent Clp protease ATP-binding subunit clpX-like, mitochondrial	292	3.70E-25	69271	19.9	0.49
Dnajc3	IPI00459033	Dnajc3 DnaJ homolog subfamily C member 3	596	1.50E-55	57428	49.6	3.24
Kdelc1	IPI00459134	Kdelc1 Isoform 1 of KDEL motif-containing protein 1	531	4.30E-49	57948	46	3.18
Sil1	IPI00338561	Sil1 Nucleotide exchange factor SIL1	421	4.60E-38	52397	29.7	1.63
Nucb1	IPI00132314	Nucb1 Nucleobindin-1	392	3.50E-35	53376	42.3	2.64
Ero1l	IPI00754386	Ero1l ERO1-like protein alpha	308	9.10E-27	54050	21.8	0.98
Dnajb11	IPI00320241	Dnajb11 DnaJ homolog subfamily B member 11	514	2.20E-47	40530	37.7	3.87
Got2	IPI00117312	Got2 Aspartate aminotransferase, mitochondrial	253	2.70E-21	47381	14.2	0.62
Actb	IPI00110850	Actb Actin, cytoplasmic 1	250	5.70E-21	41710	41.1	1.69
Wnt10a	IPI00112771	Wnt10a Protein Wnt-10a	241	5.00E-20	46424	20.6	1.21
Erp44	IPI00134058	Erp44 Endoplasmic reticulum resident protein 44	216	1.40E-17	46823	29.3	1.42
Lrpap1	IPI00469307	Lrpap1 Alpha-2-macroglobulin receptor-associated protein	203	2.60E-16	42189	31.1	1.14
Actc1	IPI00653007	Actc1 Putative uncharacterized protein	188	9.60E-15	42020	25.5	1.14
LOC100046	IPI00854971	LOC100046302 similar to Protein disulfide isomerase associated 6	169	6.70E-13	42831	21.7	0.53
Aldoa	IPI00221402	Aldoa Fructose-bisphosphate aldolase A	167	1.10E-12	39331	24.5	0.59
Calu	IPI00135186	Calu Calumenin	244	2.40E-20	37041	47	1.68
Gm5507	IPI00462075	Gm5507 similar to Glyceraldehyde-3-phosphate dehydrogenase	102	0.0000039	35771	16.7	0.67
Gm5469	IPI00461800	Gm5469 similar to heterogeneous nuclear ribonucleoprotein A3, isoform 1	101	0.0000042	37219	12.9	0.45
Idh3a	IPI00459725	Idh3a Isoform 1 of Isocitrate dehydrogenase [NAD] subunit alpha, mitochondrial	94	0.000025	39613	10.1	0.41
Cnpy3	IPI00119039	Cnpy3 Isoform 1 of Protein canopy homolog 3	287	1.10E-24	30519	26.4	2.83
LOC100042	IPI00620663	LOC100042025 similar to Glyceraldehyde-3-phosphate dehydrogenase (GAPDH) isoform 3	244	2.10E-20	31060	31.4	1.79
Capza1	IPI00330063	Capza1 F-actin-capping protein subunit alpha-1	237	1.10E-19	32919	28.7	1
LOC100047	IPI00406213	LOC100047628 similar to Chain L, Structural Basis Of Antigen Mimicry In A Clinically Relevant Melanoma Antigen System isoform 1	172	3.70E-13	26145	11.8	0.41
Necap2	IPI00133798	Necap2 Adaptin ear-binding coat-associated protein 2	157	1.00E-11	28580	19.2	0.89
Rps3a	IPI00331345	Rps3a 40S ribosomal protein S3a	126	1.50E-08	29866	20.8	0.58
Capzb	IPI00269481	Capzb Isoform 2 of F-actin-capping protein subunit beta	119	6.70E-08	30609	14.7	0.56
Cd79a	IPI00118409	Cd79a B-cell antigen receptor complex-associated protein alpha chain	100	0.0000053	24567	7.7	0.2
Eef1b2	IPI00320208	Eef1b2 Elongation factor 1-beta	89	0.000066	24678	6.7	0.2
Clpp	IPI00133270	Clpp Putative ATP-dependent Clp protease proteolytic subunit, mitochondrial	468	9.50E-43	29781	39.7	4.39
Cnpy4	IPI00875961	Cnpy4 Protein canopy homolog 4	466	1.40E-42	28076	48.2	5.99
Erp29	IPI00118832	Erp29 Endoplasmic reticulum resident protein 29	344	2.00E-30	28805	33.2	2.53
Ywhaz	IPI00116498	Ywhaz 14-3-3 protein zeta/delta	286	1.60E-24	27754	34.3	2.15
Ywhae	IPI00118384	Ywhae 14-3-3 protein epsilon	256	1.50E-21	29155	31.8	3.07
Ywhab	IPI00230682	Ywhab Isoform Long of 14-3-3 protein beta/alpha	193	2.90E-15	28069	24.8	1.25
Ywhah	IPI00227392	Ywhah 14-3-3 protein eta	191	4.30E-15	28194	27.6	1.63
Ywhaq	IPI00408378	Ywhaq Isoform 1 of 14-3-3 protein theta	185	1.90E-14	27761	21.6	0.93
Ywhag	IPI00230707	Ywhag 14-3-3 protein gamma	183	3.20E-14	28285	20.6	0.9
Igl-V2	IPI00678584	Igl-V2 LOC207685 protein (Fragment)	127	1.10E-08	24859	11.2	0.44
Mesdc2	IPI00349285	Mesdc2 LDLR chaperone MESD	335	1.80E-29	25191	42.4	2.53
Sdf2l1	IPI00227657	Sdf2l1 Stromal cell-derived factor 2-like protein 1	254	2.10E-21	23634	38	2.83
Ppic	IPI00130240	Ppic Peptidyl-prolyl cis-trans isomerase C	232	3.90E-19	22780	26.4	1.22
Ppib	IPI00135686	Ppib Peptidyl-prolyl cis-trans isomerase B	216	1.50E-17	23699	46.3	2.8

Igk-C	IPI00556847	Igk-C Igk protein	199	7.50E-16	26285	25.9	0.68
Igll1	IPI00122759	Igll1 Immunoglobulin lambda-like polypeptide 1	181	4.10E-14	22847	33	1.69
Tagln2	IPI00125778	Tagln2 Transgelin-2	165	1.70E-12	22381	42.2	2.36
Prdx1	IPI00121788	Prdx1 Peroxiredoxin-1	160	5.60E-12	22162	29.6	1.77
Cnpy2	IPI00135512	Cnpy2 Protein canopy homolog 2	146	1.50E-10	20754	28	1.39
Gstp1	IPI00555023	Gstp1 Glutathione S-transferase P 1	109	7.50E-07	23594	18.1	0.78
Manf	IPI00110350	Manf Mesencephalic astrocyte-derived neurotrophic factor	70	0.0063	20361	16.2	0.95
Hsp90b1	IPI00129526	Hsp90b1 Endoplasmin	935	1.90E-89	92418	30.9	1.86
Creld2	IPI00111286	Creld2 Cysteine-rich with EGF-like domain protein 2	216	1.30E-17	38194	11.4	1.05
Pdia6	IPI00222496	Pdia6 Putative uncharacterized protein	215	1.80E-17	48659	22.9	0.6
Rcn1	IPI00137831	Rcn1 Reticulocalbin-1	181	4.90E-14	38090	25.5	0.82
Chid1	IPI00405648	Chid1 chitinase domain-containing protein 1 isoform 2	166	1.60E-12	35518	17.4	0.67
Eef1a1	IPI00307837	Eef1a1 Elongation factor 1-alpha 1	165	1.70E-12	50082	14.7	0.73
Hspd1	IPI00308885	Hspd1 Isoform 1 of 60 kDa heat shock protein, mitochondrial	165	2.00E-12	60917	18.2	0.35
P4hb	IPI00122815	P4hb Putative uncharacterized protein	165	1.70E-12	57023	24.6	0.18
Rpsa	IPI00123604	Rpsa;LOC100045332 40S ribosomal protein SA	163	3.20E-12	32817	10.8	0.32
C3	IPI00323624	C3 Isoform Long of Complement C3 (Fragment)	162	3.30E-12	186365	3.7	0.08
Ctsd	IPI00111013	Ctsd Cathepsin D	142	3.30E-10	44925	8	0.23
Igk	IPI00462809	Igk Anti-colorectal carcinoma light chain	139	7.10E-10	26438	25.4	0.99
Ybx1	IPI00120886	Ybx1 Nuclease-sensitive element-binding protein 1	136	1.40E-09	35709	23	0.47
Txndc5	IPI00163011	Txndc5 Thioredoxin domain-containing protein 5	118	9.30E-08	46386	18.7	0.49
Set	IPI00410883	Set Isoform 2 of Protein SET	110	5.20E-07	32086	19.5	0.53
Acads	IPI00885762	Acads Protein	107	0.0000013	32593	8.6	0.15
Hnrnpd	IPI00330958	Hnrnpd Isoform 1 of Heterogeneous nuclear ribonucleoprotein D0	104	0.0000024	38330	6.5	0.27
Pdia3	IPI00230108	Pdia3 Protein disulfide-isomerase A3	102	0.0000037	56643	14.3	0.38
Pofut1	IPI00131488	Pofut1 GDP-fucose protein O-fucosyltransferase 1	101	0.000005	44660	9.4	0.23
Eif4a1	IPI00118676	Eif4a1 Eukaryotic initiation factor 4A-I	93	0.000026	46125	12.1	0.35
Tgfb1	IPI00114457	Tgfb1 Transforming growth factor beta-1	93	0.000031	44282	14.6	0.36
Ighg1	IPI00308213	Ighg1 Ig gamma-1 chain C region, membrane-bound form	92	0.000034	43359	25.7	0.24
Hnrnpab	IPI00117288	Hnrnpab Heterogeneous nuclear ribonucleoprotein A/B	86	0.00014	30812	4.9	0.16
Psmc5	IPI00135640	Psmc5 26S protease regulatory subunit 8	84	0.00021	45597	3.2	0.11
Pcbp1	IPI00128904	Pcbp1 Poly(rC)-binding protein 1	81	0.00042	37474	8.7	0.13
Fh1	IPI00129928	Fh1 Isoform Mitochondrial of Fumarate hydratase, mitochondrial	73	0.0026	54336	6.3	0.18
2210010C04	IPI00131674	2210010C04Rik trypsinogen 7	73	0.0026	26405	8.1	0.19
Htr3a	IPI00222698	Htr3a 5-hydroxytryptamine receptor 3A isoform 1	68	0.0086	56189	11	0.18
Acaa2	IPI00226430	Acaa2 3-ketoacyl-CoA thiolase, mitochondrial	67	0.012	41831	7.6	0.12
Rpl3	IPI00321170	Rpl3 60S ribosomal protein L3	62	0.035	46095	4.7	0.1
Ctse	IPI00137542	Ctse Cathepsin E	62	0.039	42905	2.5	0.11
Psmc6	IPI00125971	Psmc6 26S protease regulatory subunit S10B	61	0.041	44145	3.9	0.11
Poldip2	IPI00126634	Poldip2 Polymerase delta-interacting protein 2	192	3.80E-15	41844	13.3	0.73
Capza2	IPI00111265	Capza2 F-actin-capping protein subunit alpha-2	151	4.40E-11	32947	24.5	0.74
Mdh2	IPI00323592	Mdh2 Malate dehydrogenase, mitochondrial	129	6.40E-09	35589	33.4	0.67
Adprh	IPI00111149	Adprh [Protein ADP-ribosylarginine] hydrolase	100	0.0000054	40042	15.2	0.41
Rplp0	IPI00314950	Rplp0 60S acidic ribosomal protein P0	89	0.000077	34195	8.5	0.31
Ctso	IPI00453524	Ctso Cathepsin O	86	0.00014	34701	5.8	0.3
Eef1d	IPI00118875	Eef1d Isoform 1 of Elongation factor 1-delta	86	0.00014	31274	6.8	0.16
Tcrb-V20	IPI00130391	Tcrb-V20;Prss3;Prss1 protease, serine, 1	82	0.00038	26118	13.8	0.69
Hnrnpa2b1	IPI00405058	Hnrnpa2b1 Isoform 3 of Heterogeneous nuclear ribonucleoproteins A2/B1	79	0.00064	32440	9.3	0.53
Ctsz	IPI00125220	Ctsz Cathepsin Z	77	0.0011	34153	3.3	0.14
Npm1	IPI00127415	Npm1 Nucleophosmin	75	0.0017	32540	15.4	0.52
Hba-a1	IPI00110658	Hba-a1;Hba-a2 Putative uncharacterized protein	72	0.0033	15193	10.6	0.34
Nxph4	IPI00224986	Nxph4 Putative uncharacterized protein	71	0.0048	23109	7.9	0.22
Ldha	IPI00319994	Ldha L-lactate dehydrogenase A chain	178	9.60E-14	36475	25	0.87
Sumf2	IPI00223483	Sumf2 Sulfatase-modifying factor 2	136	1.40E-09	34685	13.3	0.3
Jup	IPI00229475	Jup Junction plakoglobin	78	0.00081	81749	5.9	0.12
Eef1a2	IPI00119667	Eef1a2 Elongation factor 1-alpha 2	74	0.0024	50422	4.1	0.2
Gm5409	IPI00395100	Gm5409 Try10-like trypsinogen	73	0.0032	26514	8.1	0.19
Pycr2	IPI00123278	Pycr2 Pyrroline-5-carboxylate reductase 2	67	0.01	33638	7.5	0.15
Cd79b	IPI00131458	Cd79b CD79B antigen	61	0.042	32113	4.9	0.15
Gm15500	IPI00308706	Gm15500;Rpl5 60S ribosomal protein L5	61	0.047	34379	4.7	0.14
Anxa2	IPI00468203	Anxa2 Annexin A2	61	0.049	38652	5.9	0.13
Pnp	IPI00315452	Pnp Purine nucleoside phosphorylase	109	6.50E-07	32256	14.9	0.53
Prdx4	IPI00116254	Prdx4 Peroxiredoxin-4	76	0.0016	31033	8.8	0.34
Etfb	IPI00121440	Etfb Electron transfer flavoprotein subunit beta	67	0.011	27606	3.9	0.18
Ccdc134	IPI00226901	Ccdc134 Isoform 1 of Coiled-coil domain-containing protein 134	64	0.023	26478	18.3	0.19
Gm13891	IPI00474637	Gm13891;Rpl10;Gm10041;LOC100048462;LOC100048223 60S ribosomal protein L10	96	0.000014	24588	6.1	0.2
Psmb1	IPI00113845	Psmb1 Proteasome subunit beta type-1	87	0.00011	26355	9.6	0.19
Gm9385	IPI00134202	Gm9385 similar to ribosomal protein L24	77	0.0011	12210	20.2	0.44
Psmb3	IPI00314467	Psmb3 Proteasome subunit beta type-3	75	0.0018	22949	6.8	0.22
Rpl23a	IPI00461456	Rpl23a;Gm10132 60S ribosomal protein L23a	74	0.0021	17684	15.4	0.29
Dnajb9	IPI00136218	Dnajb9 DnaJ homolog subfamily B member 9	70	0.0063	25720	10.4	0.42

Hspa1b	IPI00346073	Hspa1b Heat shock 70 kDa protein 1B	67	0.012	70133	4.5	0.07
Calr	IPI00123639	Calr Calreticulin	95	0.00002	47965	10.1	0.21
Igh-6	IPI00177214	Igh-6 Ig mu chain C region membrane-bound form	92	0.000039	52494	7.8	0.19
Plch1	IPI00338999	Plch1 Isoform 2 of 1-phosphatidylinositol-4,5-bisphosphate phosphodiesterase eta-1	75	0.002	182823	0.9	0.05
Prkcsh	IPI00115680	Prkcsh Isoform 1 of Glucosidase 2 subunit beta	70	0.006	58756	6	0.17
Cp	IPI00117831	Cp Ceruloplasmin	90	0.000054	121074	6.6	0.12
Srrt	IPI00224644	Srrt Isoform C of Serrate RNA effector molecule homolog	101	0.0000047	99378	8.1	0.1
Hnrnpu	IPI00458583	Hnrnpu Heterogeneous nuclear ribonucleoprotein U	196	1.60E-15	87863	11.6	0.23
Ttc13	IPI00895079	Ttc13 94 kDa protein	111	4.70E-07	93777	4	0.16
Dnajc10	IPI00229600	Dnajc10 dnaJ homolog subfamily C member 10	217	1.20E-17	90525	32.3	0.67
H6pd	IPI00222809	H6pd Hexose-6-phosphate dehydrogenase	303	2.70E-26	89853	18.2	0.51
Pkp1	IPI00124111	Pkp1 Plakophilin-1	98	0.0000092	80844	5.4	0.06
Caprin1	IPI00121515	Caprin1 caprin-1 isoform c	95	0.000017	76617	3.3	0.13
Pros1	IPI00124374	Pros1 Vitamin K-dependent protein S	110	5.30E-07	74886	7.4	0.2
Wrnip1	IPI00459468	Wrnip1 Isoform 1 of ATPase WRNIP1	303	2.60E-26	71749	23.3	0.9
Lcp1	IPI00118892	Lcp1 Plastin-2	176	1.30E-13	70105	11	0.48
Os9	IPI00230353	Os9 Isoform 2 of Protein OS-9	242	3.30E-20	69721	17.8	0.59
Ddx5	IPI00420363	Ddx5 Probable ATP-dependent RNA helicase DDX5	101	0.0000048	69277	9.4	0.31
Rpn1	IPI00309035	Rpn1 Dolichyl-diphosphooligosaccharide--protein glycosyltransferase subunit 1	62	0.034	68486	7.4	0.14
Canx	IPI00119618	Canx Calnexin	71	0.0051	67236	5.9	0.15
Tkt	IPI00137409	Tkt Transketolase	128	8.10E-09	67588	17.5	0.41
Eif3l	IPI00463573	Eif3l Eukaryotic translation initiation factor 3 subunit L	134	2.40E-09	66570	11.2	0.32
Nop56	IPI00318048	Nop56 Nucleolar protein 56	109	7.10E-07	64424	9	0.24
Fga	IPI00115522	Fga fibrinogen, alpha polypeptide isoform 2	74	0.0023	61288	4.3	0.16
Cct3	IPI00116283	Cct3 T-complex protein 1 subunit gamma	99	0.0000079	60591	16.7	0.36
4732456N1(	IPI00222228	4732456N10Rik hypothetical protein LOC239673	415	1.90E-37	58188	9.9	1.04
Plekho2	IPI00403031	Plekho2 Pleckstrin homology domain-containing family O member 2	137	1.20E-09	53839	9.5	0.41
Serpina3k	IPI00131830	Serpina3k Serine protease inhibitor A3K	238	8.40E-20	46850	26.3	0.63
Hnrnpk	IPI00223253	Hnrnpk Isoform 1 of Heterogeneous nuclear ribonucleoprotein K	187	1.20E-14	50944	22.5	0.88
Kdelc2	IPI00344686	Kdelc2 KDEL (Lys-Asp-Glu-Leu) containing 2 protein	172	3.20E-13	57650	17.1	0.38
Cct6a	IPI00116281	Cct6a T-complex protein 1 subunit zeta	131	4.10E-09	57968	13.7	0.49
Pcyox1l	IPI00226726	Pcyox1l Prenylcysteine oxidase-like	129	6.50E-09	54840	9.5	0.29
Btd	IPI00321375	Btd biotinidase	111	4.50E-07	59240	9.1	0.36
Alb	IPI00131695	Alb Serum albumin	75	0.0016	68648	10.9	0.14
C4b	IPI00131091	C4b Complement C4-B	65	0.02	192764	3.2	0.02
9530053A07	IPI00227522	9530053A07Rik Fc fragment of IgG binding protein-like	61	0.044	280044	4.3	0.02
Dsg1b	IPI00380460	Dsg1b Desmoglein-1-beta	128	8.80E-09	114382	3.7	0.22
Ckm	IPI00127596	Ckm Creatine kinase M-type	78	0.00099	43018	10.8	0.11
Pof1b	IPI00135996	Pof1b Protein POF1B	75	0.0019	68459	3.5	0.07
Aco2	IPI00116074	Aco2 Aconitate hydratase, mitochondrial	123	2.80E-08	85410	8.7	0.24
Pfkp	IPI00124444	Pfkp Isoform 1 of 6-phosphofructokinase type C	91	0.00005	85400	4.7	0.06
Aldh18a1	IPI00129350	Aldh18a1 Isoform Long of Delta-1-pyrroline-5-carboxylate synthase	85	0.00019	87242	9.8	0.17
Foxred2	IPI00132825	Foxred2 FAD-dependent oxidoreductase domain-containing protein 2	66	0.013	75956	3.3	0.06
P4ha1	IPI00272381	P4ha1 Putative uncharacterized protein	208	8.70E-17	63769	10.7	0.54
Me2	IPI00115977	Me2 NAD-dependent malic enzyme, mitochondrial	160	6.20E-12	65757	16	0.52
LOC100046	IPI00330303	LOC100046995;Atic Bifunctional purine biosynthesis protein PURH	86	0.00013	64177	6.4	0.24
Yy1	IPI00311892	Yy1 Transcriptional repressor protein YY1	85	0.00016	44689	8	0.23
Syncrip	IPI00406117	Syncrip Isoform 1 of Heterogeneous nuclear ribonucleoprotein Q	84	0.0002	69590	6.1	0.14
Sdha	IPI00230351	Sdha Succinate dehydrogenase [ubiquinone] flavoprotein subunit, mitochondrial	76	0.0013	72539	4.1	0.14
Pck2	IPI00223060	Pck2 phosphoenolpyruvate carboxykinase [GTP], mitochondrial	69	0.0066	73371	8.1	0.13
Krt18	IPI00311493	Krt18 Keratin, type I cytoskeletal 18	62	0.034	47509	7.1	0.34
Angptl2	IPI00126864	Angptl2 Angiopoietin-related protein 2	297	1.10E-25	57082	26	1.07
Tuba1a	IPI00110753	Tuba1a Tubulin alpha-1A chain	277	1.20E-23	50104	36.8	1.74
Tubb2c	IPI00169463	Tubb2c Tubulin beta-2C chain	204	2.10E-16	49799	27.4	1.09
Tuba4a	IPI00117350	Tuba4a Tubulin alpha-4A chain	199	6.70E-16	49892	38.8	1.29
Tubb2b	IPI00109061	Tubb2b Tubulin beta-2B chain	165	1.70E-12	49921	24.7	0.9
Ighg	IPI00896683	Ighg;Igh-1a;Igh-1b similar to Ig gamma-2b chain membrane isoform 1	149	6.60E-11	47138	15.8	0.34
Pkm2	IPI00407130	Pkm2 Isoform M2 of Pyruvate kinase isozymes M1/M2	120	6.20E-08	57808	18.5	0.61
Cct5	IPI00116279	Cct5 T-complex protein 1 subunit epsilon	108	8.60E-07	59586	15.7	0.36
Cct7	IPI00331174	Cct7 T-complex protein 1 subunit eta	108	9.40E-07	59614	7.4	0.26
Cct4	IPI00116277	Cct4 T-complex protein 1 subunit delta	102	0.0000038	58030	10.2	0.49
Gba	IPI00108811	Gba Glucosylceramidase	91	0.00004	57585	6.6	0.17
Gm12141	IPI00749932	Gm12141 similar to Heat shock protein 1	91	0.000045	56670	11.1	0.18
Colq	IPI00761778	Colq Acetylcholinesterase collagenic tail peptide	87	0.00012	47653	6.6	0.1
Coro1a	IPI00323600	Coro1a Coronin-1A	79	0.0008	50957	8.9	0.09

Gm10284	IPI00849049	Gm10284 similar to Glyceraldehyde-3-phosphate dehydrogenase (GAPDH) isoform 2	69	0.0074	31120	11	0.16
Fgb	IPI00279079	Fgb Fibrinogen beta chain	67	0.012	54718	11	0.29
Nap1l1	IPI00123199	Nap1l1 Nucleosome assembly protein 1-like 1	61	0.044	45317	9	0.11

S6 Table: gp96-associated proteins in preB cells. 811 clean entries were detected from Immunoprecipitation method. A total of 201 genes were selected from those 811 genes with expectation value small than 0.05.
